# Supplementary material for: Protocol for a qualitative study exploring the lived experience of hearing loss and patient reported experience in the UK: the HeLP study
Source: BMJ Open. 2023 Jun 7;13(6):e069363. doi: 10.1136/bmjopen-2022-069363 (PMC10254947; doi:10.1136/bmjopen-2022-069363)
Supplement: Supplementary data [file bmjopen-2022-069363supp001.pdf]

Aston University Birmingham

NHS University Hospitals Bristol and Weston NHS Foundation Trust

Help

# Patient/Participation Participant Information Sheet

## Hearing Loss and Patient Reported Experience (HELP) Using patient experience to improve audiology

## We need your HELP!

As someone with hearing loss, we would love your help in our study in which we are aiming to learn more about the lived experience of having hearing loss. The help we require would be in the format of an interview and questionnaire, which should take no longer than 1 hour 30 minutes. If this sounds like something you are able to do, please read on.

### What is the purpose of the study?

The aim of this study is to understand the patient's experience and the efforts people make to manage their hearing. The information you provide us with will go towards helping us create a questionnaire. This questionnaire is called a Patient Reported Experience Measure. This in turn will help us improve audiology services and improve the practical support we give people with hearing loss.

### Why have I been invited??

We would love your participation in our research, as someone seeking help with their hearing and/or experiencing hearing loss.



Pryce H, *et al. BMJ Open* 2023; 13:e069363. doi: 10.1136/bmjopen-2022-069363

## What will happen if I take part?

If you choose to take part in this study, you may be invited to participate in interviews and then invited to complete some questionnaires, including the Patient Reported Experience Measure.

We need about 30-40 people in total to participate in interviews to tell us about hearing loss. We then need a further 300 people to complete some questionnaires which are designed to capture the experience of hearing loss. Questionnaires can be completed online or on paper. We will include a stamped addressed envelope if you need to post them to us.

We may interview you again after you have completed the questionnaires to ask you what you think of the questionnaires and how we could improve them. However, as we are only recruiting a small number of people to interview after completing the questionnaire you may not be asked to take part in this. We may invite you to participate in all of these activities or you can choose one or the other.

We will conduct individual interviews at a location that suits you. That may be in person at a university or clinic premises or online or over the phone. Unfortunately, we cannot reimburse your travel expenses or time.

We will contact you using the contact details you give us e.g. phone or email.

## Do I have to take part?

No, taking part is entirely voluntary. If you decide not to take part or decide later to withdraw from the study, you won't have to give a reason and the care you receive will not be affected. You can withdraw at any time by ringing or writing to us. If you do choose to withdraw, we will only keep the data collected up to the point of withdrawal.

## Are there any risks involved?

No! Participating in the study should not cause you any inconvenience or discomfort. We are asking for your time - up to 1 hour for an interview and, on a different day, another 30 minutes for completing questionnaires.

## What are the benefits?

Your experiences are crucial to help us understand what people experience with hearing loss so we can better design the services that give support. Participating in the study will not change your clinical care right now, but might improve it in the future.

## How will we keep your data confidential?

The interviews will be recorded, and the audio files will be kept on an encrypted memory stick and a secure computer until they can be transcribed into an anonymous word document. Once it is transcribed the audio file will be deleted. No one other than the researcher and you will know that you have taken part in the study. Your clinical team will not know what you say.

## How will we use information about you?

We will need to use information from for this research project.

This information will include your name and contact details. People will use this information to do the research or to check your records to make sure that the research is being done properly. People who do not need to know who you are will not be able to see your name or contact details. Your data will have a code number instead.

We will keep all information about you safe and secure.

Once we have finished the study, we will keep some of the data so we can check the results. We will write our reports in a way that no-one can work out that you took part in the study.

## What are your choices about how your information is used?

- You can stop being part of the study at any time, without giving a reason, but we will keep information about you that we already have.



Pryce H, et al. BMJ Open 2023; 13:e069363. doi: 10.1136/bmjopen-2022-069363

- We need to manage your records in specific ways for the research to be reliable. This means that we won't be able to let you see or change the data we hold about you.

## Where can you find out more about how your information is used?

You can find out more about how we use your information

- at www.hra.nhs.uk/information-about-patients/
- our leaflet available from our website www.uhbristol.nhs.uk/research-innovation/for-patients-and-public/how-we-use-your-information-(gdpr)/
- by asking one of the research team
- by sending an email to our Data Protection Officer: InformationGovernance@UHBW.nhs.uk
- by ringing us on 0117 34 23701 or 0117 34 23794. (Data Protection Officer)

## Who is organising and funding the project?

This study has been designed by patients, audiology clinicians and researchers. The project is led by a research team at Aston University in Birmingham and the research is funded by the National Institute of Health Research (NIHR) in the Health and Social Care Delivery Research (HS&DR) Programme.

All research in the NHS is looked at by an independent group of people and this study has been reviewed and approved by the relevant Research Ethics and Health Research Authority (HRA) committees.

## What do I do now?

If you are interested in taking part in our study, please contact our research team by e-mail or telephone number asgiven below. We will then discuss whether you would like to participate in interviews and questionnaire completion. We will then arrange to meet at a time that is convenient for you.

## Who to contact for further information?

BMJ Publishing Group Limited (BMJ) disclaims all liability and responsibility arising from any reliance placed on this supplemental material which has been supplied by the author(s)


Aston University
Birmingham

NHS
University Hospitals
Bristol and Weston
NHS Foundation Trust

The Chief Investigator is Dr Helen Pryce and you can ask her any questions about the study, including any questions or concerns you may have in taking part.

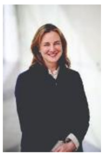

audiology_helpstudy@aston.ac.uk

0121 204 4131

**Patient Advice and Liaison Service**

If you have concerns about the way you have been approached or treated during the course of the study, you may wish to contact **Patient Advice and Liaison Service**

Kempthorne House,
St Martin's Hospital,
Midford Road,
Bath
BA2 5RPon: **t:** 01225 831500
**e:** customer services@hcrgcaregroup.com

Thank you for reading this leaflet and considering our research

HELP – Patient /Participant PIS Version 1.0 date 31/3/22 IRAS ID: 308816
