# Supplementary material for: Protocol for a qualitative study exploring the lived experience of hearing loss and patient reported experience in the UK: the HeLP study
Source: BMJ Open. 2023 Jun 7;13(6):e069363. doi: 10.1136/bmjopen-2022-069363 (PMC10254947; doi:10.1136/bmjopen-2022-069363)
Supplement: Author's manuscript [file bmjopen-2022-069363.draft_revisions.pdf]

BMJ Open

Protocol for a qualitative study exploring the lived experience of Hearing Loss and Patient Reported Experience in the UK: The HeLP study

Journal:	*BMJ Open*
Manuscript ID	bmjopen-2022-069363
Article Type:	Protocol
Date Submitted by the Author:	19-Oct-2022
Complete List of Authors:	Pryce, Helen; Aston University College of Health and Life Sciences, School of Optometry<br>Smith, Sian; Aston University College of Health and Life Sciences, School of Optometry<br>Burns-O'Connell, Georgina; Aston University College of Health and Life Sciences, School of Optometry<br>Shaw, Rachel; Aston University School of Psychology; Aston University College of Health and Life Sciences, Aston Institute of Health & Neurodevelopment<br>Hussain, Saira; Aston University College of Health and Life Sciences, Department of Audiology<br>Banks, Jonathan; National Institute for Health Research, Applied Research Collaboration West, University Hospitals Bristol and Weston NHS Foundation Trust; University of Bristol Medical School, Population Health Sciences<br>Hall, Amanda; Aston University College of Health and Life Sciences, School of Optometry; University Hospitals Bristol NHS Foundation Trust, Children's Hearing Centre<br>Knibb, Rebecca; Aston University, School of Psychology<br>Greenwood, Rosemary; University Hospitals Bristol and Weston NHS Foundation Trust<br>Straus, Jean; NIHR ARC Northwest London,
Keywords:	QUALITATIVE RESEARCH, Quality in health care < HEALTH SERVICES ADMINISTRATION & MANAGEMENT, Audiology < OTOLARYNGOLOGY

SCHOLARONE™
Manuscripts

Protocol for a qualitative study exploring the lived experience of Hearing Loss and Patient Reported Experience in the UK: The HeLP study

Authors: The HeLP project team

Dr Helen Pryce [1] *
Dr Sian K Smith [1]
Dr Georgina Burns O'Connell [1]
Dr Rachel Shaw [2, 3]
Saira Hussain [4]
Dr Jonathan Banks [5 6]
Dr Amanda Hall [1 7]
Dr Rebecca Knibb [2]
Dr Rosemary Greenwood [7]
Jean Straus [8]

Corresponding author: Dr Helen Pryce, College of Life and Health Sciences, Aston University, Birmingham, UK, B4 7ET; Email: h.pryce-cazalet@aston.ac.uk

[1] School of Optometry, College of Life and Health Sciences, Aston University, Birmingham, UK, B4 7ET
[2] School of Psychology, Aston University, Birmingham, UK, B4 7ET
[3] Institute of Health and Neurodevelopment, Aston University, Birmingham, UK, B4 7ET
[4] Department of Audiology, College of Health and Life Sciences, Birmingham, B4 7ET, UK
[5] National Institute for Health Research, Applied Research Collaboration West (NIHR ARC West), University Hospitals Bristol and Weston NHS Foundation Trust, Bristol, UK, BS1 2NT
[6] Population Health Sciences, Bristol Medical School, University of Bristol, Bristol, UK, BS8 2BN
[7] University Hospitals Bristol and Weston NHS Foundation Trust, Bristol, UK, BS1 3NU
[8] Patient and Public Involvement Representative and Lead, HeLP study, UK

For peer review only

For peer review only - http://bmjopen.bmj.com/site/about/guidelines.xhtml

ABSTRACT

Introduction

Worldwide, hearing loss is a significant public health issue and one of the most common chronic health conditions experienced by older adults. Hearing loss is associated with communication difficulties, social withdrawal, isolation, and lower quality of life. Although hearing aid technology has improved significantly, the workload of managing hearing aids has increased. The aim of this qualitative study is to develop a conceptual understanding of the lived experience of hearing loss (both with and without hearing aids) across the lifespan, and to navigate audiology services, care, and support.

Methods and analysis

Participants will be recruited from three clinical sites located in England (Bath, Bristol) and Scotland (Tayside) and non-clinical settings (e.g., lip reading classes, mosques, residential care, social media). Eligible participants will be young people and adults aged 16 years and above who have a hearing loss and carers/ family members of people with a hearing loss. This study will use individual, in-depth face-to-face or online interviews. With participants' permission, interviews will be audio-recorded and transcribed verbatim. In line with grounded theory, data collection and analysis will occur concurrently.

Ethics and dissemination

The study was approved by the West of Scotland Research Ethics Service (approval date: 6 May 2022 ref: 22/WS/0057) and the Health Research Authority and Health and Care Research Wales (HCRW) Approval (approval date:14 June 2022; IRAS project ID: 308816). The research will inform the development of a Patient Reported Experience Measure (PREM) to improve the information and support given to patients. Findings will be disseminated through peer-reviewed articles and at academic conferences, as well as to our PPI groups, healthcare professionals, audiology services and local commissioners.

Keywords: qualitative research, grounded theory, hearing loss, patient experience, hearing aids, help-seeking

For peer review only - http://bmjopen.bmj.com/site/about/guidelines.xhtml

Strengths and limitations of this study

- This protocol outlines a qualitative study exploring the lived experience of people with all types of hearing loss at different stages of life, from diverse socio-economic and cultural groups.
- This study will support conceptualisation of coping with hearing loss and navigating audiology services, as well as develop a theoretical explanation of the trade-offs that people make between using hearing aids and managing without and seeking clinical help or not.
- The conceptual model developed in this study will inform the first Patient Reported Experience Measure (PREM) internationally to gather information on patients' experiences of managing hearing loss to enable audiologists to identify the individualised and effective support.
- The theory developed in this study will be generated from lived experiences of a particular sample of people living in the UK, within a particular health and socio-political context. It is acknowledged that others may have differing experiences and this may limit the applicability of the findings, and the fit of the model to other settings.

For peer review only

For peer review only - http://bmjopen.bmj.com/site/about/guidelines.xhtml

INTRODUCTION

Hearing loss is a chronic health condition that affects an estimated 430 million adults worldwide (1). With the ageing population, hearing loss is set to affect 700 million (one in ten) of the global population by 2050. In the UK, hearing loss affects around 12 million (one in five) adults, which is predicted to increase to 14.2 million by 2035 (2). The prevalence of hearing loss rises sharply with age. In the UK, around 40% of adults aged over 50 years old live with hearing loss, increasing to 70% of older adults over the age of 70 (3). Hearing loss perpetuates socioeconomic inequality with its greatest prevalence associated with lower socioeconomic status (4, 5).

The implications of hearing loss are well-established and far reaching. People with hearing loss may feel ashamed or frustrated about their difficulty communicating and limit their social interactions (6). This separation can lead to social isolation, and feelings of loneliness, anxiety, depression, as well as fewer opportunities for engaging in listening and communicating (7-9). Hearing loss is linked with other chronic health conditions, including arthritis, cancer, cardiovascular risk factors, diabetes, stroke, visual impairment, and mobility issues (10). There is also growing evidence that hearing loss increases the risk of developing dementia, although the mechanisms linking the two are as yet unclear (11-13).

There is no permanent cure for hearing loss, and the main form of clinical intervention is wearing hearing aids. Using hearing aids has been shown to improve hearing function for mild to moderate hearing loss, as well as positively influence quality of life through enhancing communication and relationships with others (14-16). Despite these benefits, hearing aid use is low and up to 40% of people fitted with hearing aids do not wear them (17, 18). Furthermore, many people live with hearing loss symptoms for a long time (on average, up to 10 years) before they seek help (19). Understanding the reasons why hearing aid usage is low and gaining insight into why people do not seek help for hearing health (including hearing aid fitting) are important research and practice priorities.

The illness burden of hearing loss is considerable. Hearing loss makes it hard work to communicate and increases risks in all aspects of life (6, 20). Adjusting to hearing aids can be particularly tiring and frustrating in the first few months as it takes time to adapt to amplified sounds and to learn how amplified sounds correspond to previous auditory memories (21). All the treatment work is devolved to the patient, which requires considerable practical and technical work, including wearing the device each day, and replacing the fine tubing monthly, the batteries weekly, keeping the aid at the right volume settings. The stigma about visible deficits combined with the association of age with wearing hearing aids often means that people do not want hearing aids (17, 22, 23). Although hearing aid technology and assistive devices have advanced, the workload of managing hearing aids has increased significantly (e.g., learning how to use Bluetooth to pair devices, sequencing turning on and off different devices, managing complex settings) (24). Hearing aid non-use (including people who do not have hearing aids and those who have hearing aids but find it difficult to manage them) has been attributed as a problem of the hearing aid user, their motivation or ability, rather than a reflection of an

For peer review only - http://bmjopen.bmj.com/site/about/guidelines.xhtml

onerous workload. The trade-off between the benefits of wearing hearing aids versus the work required to use them is likely to vary across the life course.

The 'burden of treatment' theory is an increasingly useful way to consider the relative benefits of allocating health resources (25). Health services systematically transfer accountability and work to patients to manage long-term conditions (26, 27). Previous research has focussed on giving patients more to do, e.g. knowledge, education and skills training (28, 29) which further enhances inequality as not all adults are equally able to undertake the work required (5). Work with younger people with hearing loss transitioning into adult audiology services emphasises the burden in navigating audiology services (30). Similarly, in older age the benefits of hearing aids may be outweighed by the burden of work required to access and use them (22). This work is out of view of audiology services and thus overlooked when planning how to provide care.

In the UK, audiology services are commissioned differently in different parts of the country, with the introduction of 'Any Qualified Provider' (AQP) services meaning that large corporations and optical chains have been awarded NHS contracts to provide audiology services in England (31). AQP service providers in England are primarily funded to provide hearing aids, so there is little time (if any) to identify patients' values related to care and to engage in shared decision making about treatment options and preferences (32, 33). At the same time, guidelines from the National Institute of Clinical Excellence (NICE) propose that patients be fitted with hearing aids and then given motivational interviewing to increase their motivation to use them (34). More experiential evidence is clearly needed to understand the reasons why people do and do not use hearing aids and the factors that encourage their use.

It is critical that service development and delivery are grounded in experiential evidence to develop a good understanding of the daily illness and treatment burdens of hearing loss as well as to improve effectiveness and equity in audiology service provision. Our previous work has explored these issues from the patient perspective (22, 33). This work shows that while there are benefits in aspects of communication from hearing aids, there are specific barriers to use that derive from social identity preservation and stigma of hearing loss (and hearing aids as a visible marker of hearing loss)(20, 35). Our own work suggests that people seek help with hearing to gain information and support rather than hearing aids per se (Claesen & Pryce, 2012). However, changes in commissioning of audiology services have resulted in patients being prescribed hearing aids rather than engaging in shared decision making about their options (Pryce et al, 2016).

Further research is required to understand how people make the trade-offs at different life stages between living with a hearing loss and using a hearing aid to manage the hearing loss. Such research will enable audiology services to be sensitive to patient experience and promote shared decision making about treatment options. Therefore, the overall goal of the current study is to provide a deeper understanding of the lived experiences of coping with hearing loss (with and without hearing aids) at

For peer review only - http://bmjopen.bmj.com/site/about/guidelines.xhtml

different stages of life, from the perspectives of patients and their family & carers. This study forms part of a larger study – the Hearing Loss and Patient Reported Experience study (HeLP study)- the first study of its kind to prioritise patients' lived experience of hearing loss and treatment burden to inform decision making in audiology care planning and services. The findings from this qualitative work will inform the development and implementation of a novel Patient Reported Experience Measure (PREM) to capture the patient work involved in living with hearing loss ('illness work') and accessing and living with hearing aids ('treatment work'). We aim to identify aspects of services and treatments that are particularly challenging for patients and ultimately lead to changes in how services are delivered to improve patient experience, support, and care.

Aims and objectives

The aim of this study is to develop a comprehensive contextualised explanation of people's experiences as they live with hearing loss and the work involved in accessing and living with hearing aids. The objectives are to:

1. Conceptually explore the lived experience of coping with hearing loss at different stages of life, alongside personal and social changes, and life events.
2. Understand how individuals adapt to and manage hearing aids.
3. Explore the reasons why hearing aid non-use occurs.
4. Explore patients' experiences of accessing audiology services and support.

METHODS AND ANALYSIS

Study design

The design of this qualitative study is informed by grounded theory with the aim of developing a theoretical understanding of how coping with hearing loss ('illness work') and accessing and living with hearing aids ('treatment work') is experienced and negotiated alongside personal, cultural, and social contexts. Grounded theory methodologies are particularly well suited to developing an understanding of patterns across contrasting cases and processes that influence those patterns. In comparison to other qualitative methodologies, grounded theories offer explanatory potential as well as description (36). In this qualitative inquiry, we want to find out not only what the work of hearing loss and hearing aid use is and how it is experienced but what factors inform variation in this experience.

Study research team

The study team comprises 2 clinician-researchers and 2 researchers with complementary skills and expertise in methodology, topic, and clinical experience. The study is led and coordinated by the CI, Helen Pryce (HP), an academic and hearing therapist with 31 years of clinical experience in audiological rehabilitation. Georgina Burns O'Connell (GBOC) is a researcher with a background in

For peer review only - http://bmjopen.bmj.com/site/about/guidelines.xhtml

sociology and interest in hearing. Saira Hussain (SH) is a clinical scientist and teaching fellow in audiology with research experience. Rachel Shaw (RS) is a Health Psychologist with over 20 years of experience of conducting applied qualitative and mixed methods research in clinical settings. Sian Smith (SS) is an academic researcher with a background in health psychology, health services research and interest in hearing. Jean Straus (JS) is an expert by experience in audiology and writer.

Study steering committee group

The Study Steering Committee will have overall oversight of the study and provide advice to the study research team, funder, and sponsor of the research. Our steering group will be comprised of 8 independent members, including: 1 international collaborative representative, 2 experts by experience, 3 clinicians working in audiology in different parts of the country, 1 social science researcher and chaired by a health commissioner with a particular interest in person centred care. The steering group will meet every 6 months and oversee delivery milestones and project reporting. They will check project progress, oversee the delivery, and provide additional insight into health service context.

Study setting

The study is being conducted in the UK with participants (adults with hearing loss and carers, family members or parents) recruited from three clinical sites in England (Bristol and Bath) and Scotland (Tayside) and non-clinical groups (e.g., lip reading classes, social care settings).

Participants and recruitment

Participants are young people and adults aged 16 years and over and individuals who have experience of hearing loss directly or as a carer/ partner or parent. Eligibility criteria (Box 1) are intentionally broad to recruit participants of different ages across the life course as it is acknowledged that hearing loss experience and needs will vary at different life stages. The geographical spread of the different clinical sites (rural, urban, and semi-urban) will enable us to achieve socioeconomic variation and find cases across the age range with contrasting features in terms of sex, income, housing, and clinical needs.

Drawing on the principles of grounded theory, we will be using purposive sampling to provide maximum variation of cases to generate rich data from different backgrounds and perspectives which will allow us to target under-represented groups in the communities. We will examine the experience of different age groups and identify similarities and differences between and across the life stages This will enable us to build up an understanding of living with hearing loss (with and without hearing aids) across the life course.

Potentially eligible participants will be made aware of the study through advertisements. Clinical sites will advertise the study to new and existing patients via word-of-mouth, invitation flyers or posters displayed in waiting/reception areas. Participants will also be recruited through non-clinical routes

For peer review only - http://bmjopen.bmj.com/site/about/guidelines.xhtml

including care homes, supported living centres and community groups. Advertisements will also be distributed via leads of tinnitus groups, teachers of lip-reading classes, and staff in residential care settings. Details of the study will be circulated on social media (e.g., Twitter) and the study link hosted on Aston university website with the aim of recruiting participants with diverse experiences and backgrounds. In addition, our Personal and Public Involvement (PPI) collaborator in South Asian community groups and care homes will prompt snowball sampling in which word of mouth advertisement will encourage potential participants to contact the study team. In accordance with our PPI group in the South Asian community regarding recruitment of participants who are not help seekers, we will recruit participants from local mosques and women's groups in the local area (e.g., exercise groups for older Asian women and mindfulness group for Asian women. Previous research experience informed our sample estimate of approximately 25-38 patients and 8-12 family members/carers (see Box 1) to achieve sufficient information power, but this will be reviewed as data collection progresses.

Interested participants will directly contact the HeLP study team via email, phone, or post. The researchers will then provide potentially eligible participants with the participant information sheet (online supplemental file). Informed consent will be obtained from researchers trained in Good Clinical Practice (GCP) and experienced with research. Recruitment commenced June 2022 continuing through April 2023. At the time of writing, 18 participants have been recruited and data collection is ongoing.

Box 1. Eligibility criteria for study participation and sample

Inclusion criteria

- Young adults (aged 16-29 years): Transitioning from paediatric to adult services & negotiating independence. We will aim to recruit 4-6 young people, including those with additional disabilities such as learning disabilities, and up to 4 parents.

- Adults aged 30-49 years: Managing hearing loss while pursuing career/work and family life; 4-6 people with diagnosed hearing loss either using or not using hearing aids, plus 2-4 partners/relatives.

- Adults aged 50-79 years: Noticing symptoms of hearing loss for the first time; 4-6 hearing aid users and 5-8 non hearing aid users.

- Adults aged 80 years to end of life: Most likely to have hearing loss. Other health conditions likely and changes in living situation possible; 4-6 hearing aid users, 4-6 non hearing aid users and 2-4 carers from residential care settings.

Exclusion criteria

- Volunteers without close connection to or experience of hearing loss.

For peer review only - http://bmjopen.bmj.com/site/about/guidelines.xhtml

Data collection

Data will be collected using qualitative semi-structured individual interviews by the study team (GBOC,HP,SS,SH) who are all experienced in qualitative research methods. Participants will have the option for the interview to take place at their preferred location including online (via Microsoft Teams), or at their home, the university or audiology department in a quiet, private, and convenient space. A topic guide has been developed in accordance with the research questions exploring the lived experience of managing hearing loss and hearing service use. The topic guide has been informed by a literature review, the expertise of the authors and input from the PPI group. It covers the following topics: motivations to take part in the study, lived experience of hearing loss, challenges of hearing loss (emotions, practical efforts), the journeys and processes of referral and diagnosis, experiences of audiology services and support, decision-making around whether to wear hearing aids and trade-offs made between managing with and without hearing aids. A range of open-ended questions will be used to encourage the participant to talk in-depth and flexibly about their experiences. The expected duration of the interviews is around 1 hour. With participants' permission, the interviews will be recorded on a digital voice recorder or online using Microsoft Teams and supplemented with field notes. We will loan specialist headsets to enable people with hearing loss to hear better on the phone and via online platforms. All transcripts will be anonymised, and pseudonyms will be used.

Data analysis

In line with grounded theory methods, data analysis and interviewing will be iterative, in which early analysis and findings will inform ongoing interviews. We will keep field notes of reflections and thoughts following interviews to support this. Meaning statements will be examined and every line of transcript will be coded to summarise key content. Codes will be compared across and between transcripts and will be collated into broader categories with properties and dimensions that capture the range of meanings. Finally, codes will be grouped into a paradigm to explain the variation in experience. We will consider key themes that offer explanatory potential to the paradigm and explain variation within the data set (36). Our analysis will describe the range of 'illness work' associated with hearing loss and the 'treatment work' of using hearing aids and audiology services. We will also capture the variations that exist and the important influences that contrast different social, cultural and age groups.

At key decision making points we will consult our PPI groups (detailed below) to check that our process and early findings have not omitted any aspects of the experience. For example, during the development of topic guides, labelling codes and categories, formulating the conceptual framework, and discussing how the framework operates.

Rigour in analysis

We will ensure credibility to the research process through transparent decision making, including incorporating PPI views. The lead investigator (HP) and co-investigator (RS) will supervise the analysis closely and ensure consistency. They will blind code a subset of transcripts and compare

For peer review only - http://bmjopen.bmj.com/site/about/guidelines.xhtml

coding. The HeLP qualitative research team (GBOC, HP, SH, SS, JS) will examine and compare codes and generate a model to understand the effort required to manage hearing loss that is relevant to all age groups, as well as highlight the variations in patient need at each life stage. We will examine the fit of this model to previous models and existing theories, and we will encourage reflection from all researchers and PPI leads through discussion and debriefing.

Patient and public involvement (PPI)

Two PPI leads (a researcher with PPI responsibility and a public member) will manage and coordinate the PPI activities, working closing with the lead researcher and study team. Our PPI leads have experience in communicating with people with complex communication needs and providing support to those who have difficulties communicating and expressing preferences. PPI activities include reviewing patient information sheets and consent forms, advising on recruitment and strategy, planning interview strategy and schedules, checking codes and analysis procedures, and addressing any uncertainties in interpretation of research literature and data. Fieldnote diaries will be kept recording PPI input, activities, engagement, and outcomes.

Certain groups are more likely to be affected by hearing loss including people from South Asia (Indian, Pakistani, and Bangladeshi communities), older adults in residential care and adults with learning disabilities. Our PPI strategy reflects the diverse and heterogeneous nature of the population affected by hearing loss in the UK. We have directly targeted the following groups to expand our reach of PPI inclusion:

1. South Asian community groups – we have interest and agreement from these groups. We have also spoken to local religious centres, including but not limited to specific local Imams to enhance access to those from Muslim backgrounds.

2. Residential care homes – our PPI leads will meet with individuals in these settings (with whom we have existing relationships) face-to-face.

3. Aston PPI group – people in the local community who have experience with hearing loss, a group including student members who are younger (18-40). They meet virtually and will advise via whatever form of communication suits them best (e.g., phone, email, online messaging).

4. Bath PPI group – a well-established group of older adults who support and advise on service delivery and research. They meet face-to-face or via email.

For peer review only - http://bmjopen.bmj.com/site/about/guidelines.xhtml

ETHICS AND DISSEMINATION

Ethical approval

This study was reviewed and approved by the West of Scotland Research Ethics Service (approval date: 6 May 2022 ref: 22/WS/0057) and the Health Research Authority and Health and Care Research Wales (HCRW) Approval (approval date:14 June 2022; IRAS project ID: 308816).

Participant withdrawal

Each participant has the right to withdraw at any time during the study and information relating to all withdrawals will be recorded. If a participant wishes to discontinue, data collected up until that point will be kept and included in the analysis.

Confidentiality

All data obtained from participants will be kept confidential. Participants will be anonymised with all identifying information removed. Participants' names will be replaced with pseudonyms.

Benefits to participation

Participants may reflect on their experience of hearing loss (as a person living with hearing loss or as a carer/ family member) and gain new understandings which might be empowering. Their insights will help inform how to better design audiology services and improve clinical practice and care.

Assessment and management of risks

There is a possibility that discussing some topics will create distress. If at any point a participant feels distressed or upset during the interview, they will be given the option to pause the recording and provide time to discuss the issue. The researcher will signpost participants to other services including patient liaison services within NHS providers. In the case of home interviews, researchers will follow the sponsor's (University Hospitals Bristol and Weston NHS Trust) lone working policy including plans to check in with another member of the research team before and after interviews.

Storage of data

All study data will be stored on the university password protected server. Informed consent sheets (hard copies) containing personal information will be kept in a secure filing cabinet at the university only accessible to study staff and authorised personnel. Audio recordings of interviews will be conducted with encrypted digital recorders or MS Teams recording software. Once transcribed verbatim and checked for accuracy, recordings will be deleted. Data will be collected and retained in accordance with the Data Protection Act 2018 and General Data Protection Regulation (GDPR) standards. Once data analysis is completed, the study documents (paper and electronic) will be retained in a secure location for 5 years at Aston University.

For peer review only - http://bmjopen.bmj.com/site/about/guidelines.xhtml

For peer review only

Informed consent

We will obtain written informed consent from all interview participants at the start of an arranged interview time. Participants will have had at least a week to consider the participant information sheet and ask questions in advance. Participants who volunteer for online meetings will post their consent form back in a pre-paid envelope. The researcher will record the consent process verbally at the start of the interview as well.

Participation from adults with additional cognitive and communication difficulties are welcomed but will require capacity to consent for participation. In care home settings, where potential participants may experience dementia, researchers will check participants' capacity with care staff and only consent those who are able to provide consent independently. For this, the researcher will check that participants can understand the nature of the research, what it entails, be able to retain the information to decide about participation, be able to make a free choice, and be capable of making this decision at a time it needs to be made.

Dissemination of findings

We will prepare and publish articles in appropriate peer-reviewed journals and professional magazines (e.g., British Academy of Audiology (BAA) Audacity) to ensure the findings are shared with audiology healthcare professionals and academics researching hearing loss. Findings will be disseminated via poster and oral presentations at relevant academic and non-scientific conferences and meetings, and social media. They will also be disseminated to PPI groups, audiology services and local commissioners.

Twitter

HeLP study @HELPAstonHear

Helen Pryce @HelenPryce

Sian Smith @SianK_Smith

Georgina Burns-O'Connell @GeorgieBOC

Saira Hussain @SairaHussain90

Jean Straus @JeanStraus

Acknowledgements

The study is sponsored by University Hospitals Bristol and Weston NHS Foundation Trust and supported by Aston University Applied Audiology research group.

For peer review only

For peer review only - http://bmjopen.bmj.com/site/about/guidelines.xhtml

Funding

This study is supported by an NIHR HSDR grant (Funding stream REF NIHR 131597). The sponsor for this study is the University Hospitals Bristol and Weston NHS Foundation Trust. Aston University (Applied Audiology research group, College of Health and Life Sciences) is the supporting institution. Dr Jonathan Banks is partly funded by National Institute for Health and Care Research Applied Research Collaboration West (NIHR ARC West) and NIHR Health and Social Care Delivery HS&DR (REF NIHR 131597).

For peer review only

Competing interests: Authors have no competing interests to declare.

Patient and public involvement: Patients and/or the public were involved in the design, conduct, reporting and dissemination plans of this research. Refer to the Methods section for further information.

Authors' contributions: HP is the lead researcher leading the protocol development, ethical approval, data collection, data analysis and dissemination. SS wrote the first draft of the protocol and final manuscript. GBO, RS, SH, JB, AH, RK, RG and JS have provided feedback and support in the development of the protocol and study documents including the ethics application and interview schedule. All authors read, contributed to, edited, and agreed the final manuscript.

Patient consent for publication: N/A

Provenance and peer review: This protocol was reviewed by the South West Research Design Service Advisory Group; members of the Research Design Service; Aston University Research Governance team; PPI groups and patient experts.

ORCID IDs

Helen Pryce https://orcid.org/0000-0001-5183-6236

Sian K Smith https://orcid.org/0000-0002-9541-2221

Georgina Burns O'Connell https://orcid.org/0000-0001-6430-8627

Rachel Shaw https://orcid.org/0000-0002-0438-7666

For peer review only - http://bmjopen.bmj.com/site/about/guidelines.xhtml

Saira Hussain https://orcid.org/0000-0002-8422-1366
Jonathan Banks https://orcid.org/0000-0002-3889-6098
Amanda Hall https://orcid.org/0000-0001-8520-6005
Rebecca Knibb https://orcid.org/0000-0001-5561-0904
Jean Straus https://orcid.org/0000-0001-8023-8702

References

1. WHO. Deafness and hearing loss. https://www.who.int/news-room/fact-sheets/detail/deafness-and-hearing-loss (accessed 13 Sep 2022). 2022.
2. RNID. Hearing loss. https://rnid.org.uk/information-and-support/hearing-loss/ (accessed 13 Sep 2022). 2022.
3. Action on Hearing Loss. Hearing Matters: Why urgent action is needed on deafness, tinnitus and hearing loss across the UK. https://rnid.org.uk/about-us/research-and-policy/social-research-reports/hearing-matters/ (date accessed 17 Sep 2022). 2020.
4. Malcolm KA, Suen JJ, Nieman CL. Socioeconomic position and hearing loss: current understanding and recent advances. Current opinion in otolaryngology & head and neck surgery. 2022;30(5):351-7.
5. Scholes S, Biddulph J, Davis A, Mindell JS. Socioeconomic differences in hearing among middle-aged and older adults: cross-sectional analyses using the Health Survey for England. BMJ open. 2018;8(2):e019615.
6. Barker AB, Leighton P, Ferguson MA. Coping together with hearing loss: a qualitative meta-synthesis of the psychosocial experiences of people with hearing loss and their communication partners. International journal of audiology. 2017;56(5):297-305.
7. Chen HL. Hearing in the elderly: Relation of hearing loss, loneliness, and self-esteem. Journal of Gerontological Nursing. 1994;20(6):22-8.
8. Shukla A, Harper M, Pedersen E, Goman A, Suen JJ, Price C, et al. Hearing Loss, Loneliness, and Social Isolation: A Systematic Review. Otolaryngology--head and neck surgery : official journal of American Academy of Otolaryngology-Head and Neck Surgery. 2020;162(5):622-33.
9. Lawrence BJ, Jayakody DMP, Bennett RJ, Eikelboom RH, Gasson N, Friedland PL. Hearing Loss and Depression in Older Adults: A Systematic Review and Meta-analysis. The Gerontologist. 2020;60(3):e137-e54.
10. Besser J, Stropahl M, Urry E, Launer S. Comorbidities of hearing loss and the implications of multimorbidity for audiological care. Hearing Research. 2018;369:3-14.
11. Lin FR, Metter EJ, O'Brien RJ, Resnick SM, Zonderman AB, Ferrucci L. Hearing Loss and Incident Dementia. Archives of Neurology. 2011;68(2):214-20.

For peer review only

For peer review only - http://bmjopen.bmj.com/site/about/guidelines.xhtml

12. Loughrey DG, Kelly ME, Kelley GA, Brennan S, Lawlor BA. Association of Age-Related Hearing Loss With Cognitive Function, Cognitive Impairment, and Dementia: A Systematic Review and Meta-analysis. JAMA Otolaryngology–Head & Neck Surgery. 2018;144(2):115-26.
13. Griffiths TD, Lad M, Kumar S, Holmes E, McMurray B, Maguire EA, et al. How Can Hearing Loss Cause Dementia? Neuron. 2020;108(3):401-12.
14. Ferguson MA, Kitterick PT, Chong LY, Edmondson-Jones M, Barker F, Hoare DJ. Hearing aids for mild to moderate hearing loss in adults. Cochrane Database of Systematic Reviews. 2017(9).
15. Kitterick PT, Ferguson MA. Hearing Aids and Health-Related Quality of Life in Adults With Hearing Loss. JAMA. 2018;319(21):2225-6.
16. Chisolm TH, Johnson CE, Danhauer JL, Portz LJ, Abrams HB, Lesner S, et al. A systematic review of health-related quality of life and hearing aids: final report of the American Academy of Audiology Task Force On the Health-Related Quality of Life Benefits of Amplification in Adults. Journal of the American Academy of Audiology. 2007;18(2):151-83.
17. McCormack A, Fortnum H. Why do people fitted with hearing aids not wear them? International journal of audiology. 2013;52(5):360-8.
18. Knudsen LV, Oberg M, Nielsen C, Naylor G, Kramer SE. Factors influencing help seeking, hearing aid uptake, hearing aid use and satisfaction with hearing aids: a review of the literature. Trends in amplification. 2010;14(3):127-54.
19. Davis A, Smith P, Ferguson M, Stephens D, Gianopoulos I. Acceptability, benefit and costs of early screening for hearing disability: a study of potential screening tests and models. Health technology assessment (Winchester, England). 2007;11(42):1-294.
20. Holman JA, Ali YHK, Naylor G. A qualitative investigation of the hearing and hearing-aid related emotional states experienced by adults with hearing loss. International journal of audiology. 2022:1-10.
21. Wright D, Gagné JP. Acclimatization to Hearing Aids by Older Adults. Ear Hear. 2021;42(1):193-205.
22. Claesen E, Pryce H. An exploration of the perspectives of help-seekers prescribed hearing aids. Primary health care research & development. 2012;13(3):279-84.
23. Humes LE, Wilson DL, Barlow NN, Garner C. Changes in hearing-aid benefit following 1 or 2 years of hearing-aid use by older adults. Journal of speech, language, and hearing research : JSLHR. 2002;45(4):772-82.
24. Stieglitz Ham H, Bunn P, Meyer C, Khan A, Hickson L. Exploring the relationship between technology use, hearing help-seeking, and hearing aid outcomes in older adults. International journal of audiology. 2014;53 Suppl 1:S38-42.
25. May CR, Eton DT, Boehmer K, Gallacher K, Hunt K, MacDonald S, et al. Rethinking the patient: using Burden of Treatment Theory to understand the changing dynamics of illness. BMC Health Services Research. 2014;14(1):281.
26. Gallacher K, May CR, Montori VM, Mair FS. Understanding patients' experiences of treatment burden in chronic heart failure using normalization process theory. Annals of family medicine. 2011;9(3):235-43.

For peer review only - http://bmjopen.bmj.com/site/about/guidelines.xhtml

27. Jowsey T, Yen L, W PM. Time spent on health related activities associated with chronic illness: a scoping literature review. BMC Public Health. 2012;12(1):1044.

28. Ferguson M, Brandreth M, Brassington W, Leighton P, Wharrad H. A Randomized Controlled Trial to Evaluate the Benefits of a Multimedia Educational Program for First-Time Hearing Aid Users. Ear Hear. 2016;37(2):123-36.

29. Sweetow RW, Sabes JH. The need for and development of an adaptive Listening and Communication Enhancement (LACE) Program. Journal of the American Academy of Audiology. 2006;17(8):538-58.

30. Spyridakou C, Mendis S, DeVal D. Improving transition outcomes in adolescents with permanent hearing loss. BMJ Open Quality. 2019;8(2):e000336.

31. Department of Health. Operational Guidance to the NHS: extending patient choice of provider. Gateway ref 16242. 2011.

32. Pryce H, Hall A. The role of shared decision-making in audiologic rehabilitation. Perspectives on Aural Rehabilitation and Its Instrumentation. 2014;21(1):15-23.

33. Pryce H, Hall A, Laplante-Lévesque A, Clark E. A qualitative investigation of decision making during help-seeking for adult hearing loss. International journal of audiology. 2016;55(11):658-65.

34. NICE. Hearing loss in adults: assessment and management. NICE guideline Published: 21 June 2018 www.nice.org.uk/guidance/ng98 (accessed 12 Sep 2022). 2018.

35. Barker AB, Leighton P, Ferguson MA. Coping together with hearing loss: a qualitative meta-synthesis of the psychosocial experiences of people with hearing loss and their communication partners. International journal of audiology. 2017;56(5):297-305.

36. Corbin J SA. Basics of qualitative research: Techniques and procedures for developing grounded theory. Sage publications; 2014 Nov 25. 2014.

For peer review only

For peer review only - http://bmjopen.bmj.com/site/about/guidelines.xhtml

Protocol for a qualitative study exploring the lived experience of Hearing Loss and Patient Reported Experience in the UK: The HeLP study

Journal:	*BMJ Open*
Manuscript ID	bmjopen-2022-069363.R1
Article Type:	Protocol
Date Submitted by the Author:	17-Mar-2023

Complete List of Authors:	Pryce, Helen; Aston University College of Health and Life Sciences, School of Optometry<br>Smith, Sian; Aston University College of Health and Life Sciences, School of Optometry<br>Burns-O'Connell, Georgina; Aston University College of Health and Life Sciences, School of Optometry<br>Shaw, Rachel; Aston University School of Psychology; Aston University College of Health and Life Sciences, Aston Institute of Health & Neurodevelopment<br>Hussain, Saira; Aston University College of Health and Life Sciences, Department of Audiology<br>Banks, Jonathan; National Institute for Health Research, Applied Research Collaboration West, University Hospitals Bristol and Weston NHS Foundation Trust; University of Bristol Medical School, Population Health Sciences<br>Hall, Amanda; Aston University College of Health and Life Sciences, School of Optometry; University Hospitals Bristol NHS Foundation Trust, Children's Hearing Centre<br>Knibb, Rebecca; Aston University, School of Psychology<br>Greenwood, Rosemary; University Hospitals Bristol and Weston NHS Foundation Trust<br>Straus, Jean; NIHR ARC Northwest London,

**Primary Subject Heading**:	Qualitative research
Secondary Subject Heading:	Health services research
Keywords:	QUALITATIVE RESEARCH, Quality in health care < HEALTH SERVICES ADMINISTRATION & MANAGEMENT, Audiology < OTOLARYNGOLOGY, Patient-Centered Care

For peer review only - http://bmjopen.bmj.com/site/about/guidelines.xhtml

Protocol for a qualitative study exploring the lived experience of Hearing Loss and Patient Reported Experience in the UK: The HeLP study

Authors: The HeLP project team

Dr Helen Pryce [1] *
Dr Sian K Smith [1]
Dr Georgina Burns O'Connell [1]
Dr Rachel Shaw [2, 3]
Saira Hussain [4]
Dr Jonathan Banks [5 6]
Dr Amanda Hall [1 7]
Dr Rebecca Knibb [2]
Dr Rosemary Greenwood [7]
Jean Straus [8]

Corresponding author: Dr Helen Pryce, College of Life and Health Sciences, Aston University, Birmingham, UK, B4 7ET; Email: h.pryce-cazalet@aston.ac.uk

[1] School of Optometry, College of Life and Health Sciences, Aston University, Birmingham, UK, B4 7ET
[2] School of Psychology, Aston University, Birmingham, UK, B4 7ET
[3] Institute of Health and Neurodevelopment, Aston University, Birmingham, UK, B4 7ET
[4] Department of Audiology, College of Health and Life Sciences, Birmingham, B4 7ET, UK
[5] National Institute for Health Research, Applied Research Collaboration West (NIHR ARC West), University Hospitals Bristol and Weston NHS Foundation Trust, Bristol, UK, BS1 2NT
[6] Population Health Sciences, Bristol Medical School, University of Bristol, Bristol, UK, BS8 2BN
[7] University Hospitals Bristol and Weston NHS Foundation Trust, Bristol, UK, BS1 3NU
[8] Patient and Public Involvement Representative and Lead, HeLP study, UK

For peer review only

For peer review only - http://bmjopen.bmj.com/site/about/guidelines.xhtml

ABSTRACT

Introduction

Worldwide, hearing loss is a significant public health issue and one of the most common chronic health conditions experienced by older adults. Hearing loss is associated with communication difficulties, social withdrawal, isolation, and lower quality of life. Although hearing aid technology has improved significantly, the workload of managing hearing aids has increased. The aim of this qualitative study is to develop a theoretical understanding of the lived experience of hearing loss (both with and without hearing aids) across the lifespan, and to navigate audiology services.

Methods

Eligible participants will be young people and adults aged 16 years and above who have a hearing loss and carers/ family members of people with a hearing loss. This study will use individual, in-depth face-to-face or online interviews. With participants' permission, interviews will be audio-recorded and transcribed verbatim. A grounded theory approach to concurrent data gathering and analysis will develop grouped codes and categories and link these to provide a novel theory to describe the experience of hearing loss.

Ethics and dissemination

The study was approved by the West of Scotland Research Ethics Service (approval date: 6 May 2022 ref: 22/WS/0057) and the Health Research Authority and Health and Care Research Wales (HCRW) Approval (approval date:14 June 2022; IRAS project ID: 308816). The research will inform the development of a Patient Reported Experience Measure (PREM) to improve the information and support given to patients. Findings will be disseminated through peer-reviewed articles and at academic conferences, as well as to our Patient and Public Involvement (PPI) groups, healthcare professionals, audiology services and local commissioners.

Keywords: qualitative research, grounded theory, hearing loss, patient experience, hearing aids, help-seeking

Word count: 3994

For peer review only

Strengths and limitations of this study

- Our qualitative interview study, informed by grounded theory approaches, will generate a novel theory on the lived experience of hearing loss across the life course.
- Different patient, public involvement (PPI) groups will be involved throughout the project to guide the logistics of conducting the research and support data analysis procedures.
- Purposive sampling via different clinical sites and non-clinical routes (e.g., lip reading classes, social media) will help to ensure diverse experiences and views are captured.
- The study will be restricted to people living in the UK which may limit the applicability of the findings to other settings.
- This qualitative study part of a wider study that will inform the development of the first Patient Reported Experience Measure (PREM) internationally to enable audiologists to identify the individualised and effective support.

For peer review only

INTRODUCTION

Hearing loss is a chronic health condition that affects an estimated 430 million adults worldwide (1). With the ageing population, hearing loss is set to affect 700 million (one in ten) of the global population by 2050. In the UK, hearing loss affects around 12 million (one in five) adults, which is predicted to increase to 14.2 million by 2035 (2). The prevalence of hearing loss rises sharply with age. In the UK, around 40% of adults aged over 50 years old live with hearing loss, increasing to 70% of older adults over the age of 70 (3). Hearing loss perpetuates socioeconomic inequality with its greatest prevalence associated with lower socioeconomic status (4, 5).

The implications of hearing loss are well-established and far reaching. People with hearing loss may feel ashamed or frustrated about their difficulty communicating and limit their social interactions (6). This separation can lead to social isolation, and feelings of loneliness, anxiety, depression, as well as fewer opportunities for engaging in listening and communicating (7-9). Hearing loss is linked with other chronic health conditions, including arthritis, cancer, cardiovascular risk factors, diabetes, stroke, visual impairment, and mobility issues (10). There is also growing evidence that hearing loss increases the risk of developing dementia, although the mechanisms linking the two are as yet unclear (11-13).

There is no permanent cure for hearing loss, and the main form of clinical intervention is wearing hearing aids. Using hearing aids has been shown to improve hearing function for mild to moderate hearing loss, as well as positively influence quality of life through enhancing communication and relationships (14-16). Despite these benefits, hearing aid use is low and approximately 30% of people report wearing hearing aids some of the time and 20% do not wear them at all (17). Furthermore, many people live with hearing loss symptoms for a long time (on average, up to 10 years) before seeking help (18). Understanding the reasons why hearing aid usage is low and gaining insight into why people do not seek help are important research priorities.

The illness burden of hearing loss is considerable. Hearing loss makes it hard work to communicate and increases risks in all aspects of life (6, 19). Adjusting to hearing aids can be particularly tiring and frustrating as it takes time to adapt to amplified sounds and to learn how amplified sounds correspond to previous auditory memories (20). All the treatment work is devolved to the patient, including wearing the device each day, and replacing the fine tubing monthly, the batteries weekly, keeping the aid at the right volume settings. The stigma about visible deficits combined with the association of age with wearing hearing aids often means that people do not want hearing aids (21-23). Although hearing aid technology and assistive devices have advanced, the workload of managing hearing aids has increased significantly (e.g., learning how to use Bluetooth to pair devices, sequencing turning on and off different devices) (24). Hearing aid non-use (including people who do not have hearing aids and those who have hearing aids but find it difficult to manage them) has been attributed as a problem of the hearing aid user, their motivation or ability, rather than a reflection of an onerous workload. The

For peer review only - http://bmjopen.bmj.com/site/about/guidelines.xhtml

trade-off between the benefits of wearing hearing aids versus the work required to use them is likely to vary across the life course.

The 'burden of treatment' theory is an increasingly useful way to consider the relative benefits of allocating health resources (25). Health services systematically transfer accountability and work to patients to manage long-term conditions (26, 27). Previous research has focussed on giving patients more to do, e.g. education and skills training (28, 29) which further enhances inequality as not all adults are equally able to undertake the work required (5). Work with younger people with hearing loss transitioning into adult audiology services emphasises the burden in navigating audiology services (30). Similarly, in older age the benefits of hearing aids may be outweighed by the burden of work required to access and use them (21). This work is out of view of audiology services and thus overlooked when planning how to provide care.

In the UK, audiology services are commissioned differently in different parts of the country, with the introduction of 'Any Qualified Provider' (AQP) services meaning that large corporations and optical chains have been awarded NHS contracts to provide audiology services in England (31). AQP service providers are primarily funded to provide hearing aids, so there is little time (if any) to identify patients' values related to care and to engage in shared decision making about treatment options and preferences (32, 33). At the same time, guidelines from the National Institute of Clinical Excellence (NICE) propose that patients be fitted with hearing aids and then given motivational interviewing to increase their motivation to use them (34). More experiential evidence is needed to understand the reasons why people do and do not use hearing aids and the factors that encourage their use.

It is critical that service development and delivery are grounded in experiential evidence to develop a good understanding of the daily illness and treatment burdens of hearing. Previous work shows that while there are benefits in aspects of communication from hearing aids, there are specific barriers to use that derive from social identity preservation and stigma of hearing loss (and hearing aids as a visible marker of hearing loss) (19, 21, 33, 35). Our own work suggests that people seek help with hearing to gain information and support rather than hearing aids per se (Claesen & Pryce, 2012). However, changes in commissioning of audiology services have resulted in patients being prescribed hearing aids rather than engaging in shared decision making (Pryce et al, 2016).

Further research is required to understand how people make the trade-offs at different life stages between living with a hearing loss and using a hearing aid to manage the hearing loss. Such research will enable audiology services to be sensitive to patient experience and promote shared decision making about options. Therefore, the overall goal of the current study is to provide a deeper understanding of the lived experiences of coping with hearing loss (with and without hearing aids) at different stages of life, from the perspectives of patients and their families. This study forms part of a larger study – the Hearing Loss and Patient Reported Experience study (HeLP study) –

For peer review only - http://bmjopen.bmj.com/site/about/guidelines.xhtml

the first study of its kind to prioritise patients' lived experience of hearing loss to inform decision-making in audiology care planning and services. The findings from this qualitative work will inform the development of potential items for a novel Patient Reported Experience Measure (PREM) to be used in clinical practice to understand the work involved in living with hearing loss ('illness work') and hearing aids ('treatment work').

Aims and objectives

Drawing on grounded theory methodology, the aim of this study is to develop a comprehensive novel theory that has the potential to explain variation in people's lived experiences of hearing loss. The objectives are to:

1. Develop a theoretical understanding of how coping with hearing loss ('illness work') and accessing and living with hearing aids ('treatment work') is experienced and negotiated alongside personal and socio-cultural contexts.
2. Understand how individuals adapt to and manage hearing aids.
3. Explore the reasons why hearing aid non-use occurs.
4. Explore patients' experiences of accessing audiology services and support.

METHODS AND ANALYSIS

Study design

This qualitative interview study will be informed by Strauss and Corbin's pragmatic approach to grounded theory (36, 37). This allows for (and recognises the likelihood of) researchers to bringing prior knowledge to the process of refining theoretical concepts. Grounded theory methodologies are particularly well suited to developing an understanding of patterns across contrasting cases and processes that influence those patterns. In comparison to other qualitative methodologies, grounded theories offer explanatory potential as well as description (38). In this work, we want to identify the work involved in hearing loss and hearing aid use, how it is experienced, and the factors informing variation in this experience.

Research team and reflexivity

For reflexivity, the study team comprises two female clinician-researchers (HP – an academic and hearing therapist with clinical experience in audiological rehabilitation and grounded theory methodology; SH – a clinical scientist, teaching fellow and researcher in audiology), three female researchers (GBOH – a teaching fellow and researcher with a background in sociology and interest in hearing research; RS – a Health Psychologist with experience of applied qualitative research; SS – a Health Psychologist with personal interest in hearing research) and a female PPI lead (JS) who is an expert in audiology by experience and writer. All the team either have first-hand experience of hearing loss or are closely related to someone who does.

For peer review only

Participants will be interviewed by either HP, GBOC, SH or SS. At the start of each interview, the interviewers will undertake a ‘working alliance’ discussion agreeing participant and researcher roles, timing and nature of the interview. This will include the researcher’s interest and motivations in the topic.

The study team will meet fortnightly to reflect on their role in shaping data collection and compare and contrast their interpretations of the data. Throughout the study, the wider HeLP research group and core PPI members will discuss interpretations of the findings for triangulation and work together to develop and link theoretical categories for the explanatory model.

Study steering committee group

The Study Steering Committee will have overall oversight of the study and provide advice to the study research team, funder, and sponsor. Our steering group will comprise 8 independent members, including: 1 international collaborative representative, 2 experts by experience, 3 clinicians working in audiology in different parts of the country, 1 social science researcher and chaired by a health commissioner with a particular interest in person centred care. The steering group will meet every 6 months and oversee delivery milestones and project reporting.

Study setting

The study is being conducted in the UK with participants (adults with hearing loss and carers, family members or parents) recruited from three clinical sites in England (Bristol and Bath) and Scotland (Tayside) and non-clinical groups (e.g., lip reading classes, social care settings).

Participants and recruitment

Participants are young people and adults aged 16 years and over and individuals who have experience of hearing loss directly or as a carer, partner or parent. Eligibility criteria (Box 1) are intentionally broad to recruit participants of different ages across the life course as it is acknowledged that hearing loss experience and needs will vary at different life stages. The geographical spread of the different clinical sites (rural, urban, and semi-urban) will enable us to achieve socioeconomic variation and find cases across the age range with contrasting features in terms of sex, income, housing, and clinical needs.

Drawing on the principles of Strass and Corbin’s grounded theory approach, we will be using purposive sampling to provide maximum variation of cases to generate rich data from different perspectives which will allow us to target under-represented groups. We will examine the experience of different age groups and identify similarities and differences between and across the life stages This will enable us to build up an understanding of living with hearing loss (with and without hearing aids) across the life course.

For peer review only

For peer review only - http://bmjopen.bmj.com/site/about/guidelines.xhtml

Potentially eligible participants will be made aware of the study through advertisements. Clinical sites will advertise the study to new and existing patients via word-of-mouth, invitation flyers or posters displayed in waiting/reception areas. Participants will also be recruited through non-clinical routes including care homes, supported living centres and community groups. Advertisements will also be distributed via leads of tinnitus groups, teachers of lip-reading classes, and staff in residential care settings. Details of the study will be circulated on social media (e.g., Twitter) and the study link hosted on Aston university website with the aim of recruiting participants with diverse experiences and backgrounds. In addition, our Patient and Public Involvement (PPI) collaborator in South Asian community groups and care homes will prompt snowball sampling in which word of mouth advertisement will encourage potential participants to contact the study team. In accordance with our PPI group in the South Asian community regarding recruitment of participants who are not help seekers, we will recruit participants from local mosques and women's groups in the local area (e.g., exercise groups for older Asian women and mindfulness group for Asian women. Previous research experience informed our sample estimate of approximately 25-38 patients and 8-12 family members/carers (see Box 1) to capture a range of experiences and achieve sufficient information power, but this will be reviewed as data collection progresses.

Interested participants will directly contact the HeLP study team via email, phone, or post. The researchers will then provide potentially eligible participants with the participant information sheet (online supplemental file). Informed consent will be obtained from researchers trained in Good Clinical Practice (GCP) and experienced with research. Recruitment commenced June 2022 continuing through April 2023. At the time of writing, 18 participants have been recruited and data collection is ongoing.

Box 1. Eligibility criteria for study participation and sample

Inclusion criteria

- Young adults (aged 16-29 years): Transitioning from paediatric to adult services & negotiating independence. We will aim to recruit 4-6 young people, including those with additional disabilities such as learning disabilities, and up to 4 parents.
- Adults aged 30-49 years: Managing hearing loss while pursuing career/work and family life; 4-6 people with diagnosed hearing loss either using or not using hearing aids, plus 2-4 partners/relatives.
- Adults aged 50-79 years: Noticing symptoms of hearing loss for the first time; 4-6 hearing aid users and 5-8 non hearing aid users.
- Adults aged 80 years to end of life: Most likely to have hearing loss. Other health conditions likely and changes in living situation possible; 4-6 hearing aid users, 4-6 non hearing aid users and 2-4 carers from residential care settings.

For peer review only - http://bmjopen.bmj.com/site/about/guidelines.xhtml

Exclusion criteria

- Volunteers without close connection to or experience of hearing loss.

Data collection

Data will be collected using qualitative, semi-structured interviews by the study team (GBOC,HP, SS,SH) who are all experienced in qualitative research. Participants will have the option for the interview to take place at their preferred location including online (via Microsoft Teams), or at their home, the university or audiology department in a quiet and private space. An interview schedule been developed to explore the lived experience of managing hearing loss and hearing service use (Box 2). It has been informed by a literature review, the expertise of the authors and input from the PPI groups. It covers: motivations to take part in the study, lived experience of hearing loss, challenges of hearing loss (emotions, practical efforts), the journeys and processes of referral and diagnosis, experiences of audiology services and support, decision-making around whether to wear hearing aids and trade-offs made between managing with and without hearing aids. Open-ended questions will be used to encourage the participant to talk in-depth and flexibly about their experiences (Box 2). The expected duration of the interviews is around 1 hour. With participants' permission, the interviews will be recorded on a digital voice recorder or online (video and audio-recorded) using Microsoft Teams and supplemented with field notes to capture important information not captured by the audio-recording. We will loan specialist headsets to enable people with hearing loss to hear better on the phone and via online platforms. All transcripts will be anonymised, and pseudonyms will be used. Transcripts will not be returned to participants for comment and/or correction as we want to capture experiences and views at that particular moment in time.

Box 2. Interview questions for patients and carers/ family members

Questions for people with hearing loss

- Tell me a little bit about yourself
- Tell me your story with your hearing and why you were interested in taking part?
- Can you tell me your thoughts and feelings about hearing loss?
- What have you found difficult about having a hearing loss?
- What have you done to manage your hearing loss?
- Decision making around hearing aid use or non-use/ uptake – Tell me how you came to be using/not using your assistive listening devices/hearing aids explore reasons why/why not?
- What is important to you when deciding whether to use hearing devices/aids?
- Who else is important in helping you decide?
- Has this changed over time?
- What has been helpful to you?
- Have you sought help from audiology services?
- Tell me about your experience with using audiology services
- Based on your experience – what do you think they ought to know/ do that they currently don't do?

Questions for family/ carers

- Tell me what is it like for you managing hearing loss?
- What is difficult for you about living with someone with hearing loss?
- What if anything do you do differently as a result of hearing loss?

For peer review only - http://bmjopen.bmj.com/site/about/guidelines.xhtml

- Have you had any involvement with audiology services?
- What was that like?
- What is challenging about using earing aids (if your family member/cared for person has them)?

Data analysis

In line with Strauss and Corbin's grounded theory procedures, data analysis and interviewing will be iterative, in which early analysis and findings will be compared to inform ongoing interviews. We will keep field notes of reflections and thoughts following interviews to support this. Whilst the notion of data 'saturation' has been widely critiqued as a neo-positivist indication of a singular 'truth'; there is recognition of the importance of capturing a range of views, based on different contexts (39). The research team will review the data until we are confident that a wide range of experiences are captured and there is sufficient conceptual depth to generate meaningful insights to address the research aims (39). Meaning statements will be examined and every line of transcript will be coded by three coders (GBOC, SH, SS) to summarise key content (open coding). Codes will be compared across and between transcripts and will be collated into broader categories with properties and dimensions that capture the range of meanings (axial coding). Finally, the relationships between these categories will be explored to develop a conceptual model that explains the variation in experience (selective coding). Triangulation is a core part of our activity with multiple researcher and PPI members contributing a view to the interpretation which is justified by each researcher. We will consider core categories that offer explanatory potential to the paradigm and explain variation within the data set (38). Our analysis will describe the range of 'illness work' of hearing loss and the 'treatment work' of using hearing aids and audiology services. We will also capture the variations that exist and the important influences that contrast different social, cultural and age groups.

Rigour in analysis

We will ensure credibility to the research process through transparent decision making, including incorporating PPI views. The lead investigator (HP) and co-investigator (RS) will supervise the analysis closely and ensure consistency. They will blind code a subset of transcripts and compare coding. The HeLP qualitative research team (GBOC, HP, SH, JS, SS) will develop and refine the conceptual model whilst interviews and analysis progress to understand the effort required to manage hearing loss and highlight the variations in patient need at each life stage. We will examine the fit of this model to previous models and existing theories, and we will encourage reflection from all researchers and PPI leads through discussion and debriefing.

Patient and public involvement (PPI)

A major component of the HeLP study is the involvement of PPI groups. Specifically, two PPI leads (a researcher with PPI responsibility and a public member) will manage and coordinate the PPI activities, working closing with the study team. Our PPI leads have experience in communicating with people with complex communication needs. PPI activities include reviewing patient information sheets and consent forms, advising on recruitment and interview questions, checking analysis procedures,

For peer review only - http://bmjopen.bmj.com/site/about/guidelines.xhtml

and addressing any uncertainties in data interpretation We will consult our PPI groups to check that our process and early findings have not omitted any aspects of the experience (e.g. labelling codes and categories, development of conceptual framework). Fieldnote diaries will record PPI engagement and outcomes.

Certain groups are more likely to be affected by hearing loss including people from South Asia (Indian, Pakistani, and Bangladeshi communities), older adults in residential care and adults with learning disabilities. Our PPI strategy reflects the diverse and heterogeneous nature of the population affected by hearing loss in the UK. We have directly targeted the following groups to expand our reach of PPI inclusion:

1. South Asian community groups – we have interest and agreement from these groups. We have also spoken to local religious centres, including but not limited to specific local Imams to enhance access to those from Muslim backgrounds. South Asian heritage people have both a disproportionately high risk of hearing loss and are underrepresented in patient populations in the UK (40, 41).

2. Residential care homes – our PPI leads will meet with individuals in these settings (with whom we have existing relationships) face-to-face.

3. Aston PPI group – people in the local community who have experience with hearing loss, a group including student members who are younger (18-40). They meet virtually and will advise via whatever form of communication suits them best (e.g., phone, email, online messaging).

4. Bath PPI group – a well-established group of older adults who support and advise on service delivery and research. They meet face-to-face or via email.

Our initial PPI work informed the development of the proposal in a number of ways. Firstly, our Aston group highlighted the need for researchers from the same culture and religious as participants to undertake PPI and research. As such, we will include a researcher with responsibility for PPI in marginalised groups and have recruited a researcher dedicated to South Asian groups. Secondly, our older group identified problems with hearing aids that inform the notion of 'work' (e.g. struggles to fit hearing aids, learning skills such as battery changing). These issues will be explored further by targeting PPI and recruitment in residential care settings where access to audiology services can be challenging. Thirdly, our older group noted the need to be flexible in where and how data gathering occurs and be mindful of travel and distance barriers for participants. This informed our plans to gather data in people's homes, at clinics, university, and online/ telephone. Finally, our younger group advised we purchase amplifier headsets which could be sent out to participants for online/phone use, if required.

For peer review only - http://bmjopen.bmj.com/site/about/guidelines.xhtml

ETHICS AND DISSEMINATION

Ethical approval

This study was reviewed and approved by the West of Scotland Research Ethics Service (approval date: 6 May 2022 ref: 22/WS/0057) and the Health Research Authority and Health and Care Research Wales (HCRW) Approval (approval date:14 June 2022; IRAS project ID: 308816).

Participant withdrawal

Each participant has the right to withdraw at any time during the study and information relating to all withdrawals will be recorded. If a participant wishes to discontinue, data collected up until that point will be kept and included in the analysis.

Confidentiality

All data obtained from participants will be kept confidential. Participants will be anonymised with all identifying information removed. Participants' names will be replaced with pseudonyms.

Benefits to participation

Participants may reflect on their experience of hearing loss (as a person living with hearing loss or as a carer/ family member) and gain new understandings which might be empowering. Their insights will help inform how to better design audiology services and improve clinical practice and care.

Assessment and management of risks

There is a possibility that discussing some topics will create distress. If at any point a participant feels distressed or upset during the interview, they will be given the option to pause the recording and provide time to discuss the issue. The researcher will signpost participants to other services including patient liaison services within NHS providers. In the case of home interviews, researchers will follow the sponsor's (University Hospitals Bristol and Weston NHS Trust) lone working policy including plans to check in with another member of the research team before and after interviews.

Storage of data

All study data will be stored on the university password protected server. Informed consent sheets (hard copies) containing personal information will be kept in a secure filing cabinet at the university only accessible to study staff and authorised personnel. Audio recordings of interviews will be conducted with encrypted digital recorders or MS Teams recording software. Once transcribed verbatim and checked for accuracy, recordings will be deleted. Data will be collected and retained in accordance with the Data Protection Act 2018 and General Data Protection Regulation (GDPR) standards. Once data analysis is completed, the study documents (paper and electronic) will be retained in a secure location for 5 years at Aston University. Personal data (e.g. contact details) are held on a separate Aston University Box file and are separate from research data. Besides clinicians

For peer review only

in Participant Identification Centres (PICs) sending the advertisements to home addresses we will not access personal data.

Informed consent

We will obtain written informed consent from all interview participants at the start of an arranged interview time. Participants will have had at least a week to consider the participant information sheet and ask questions in advance. Participants who volunteer for online meetings will post their consent form back in a pre-paid envelope. The researcher will record the consent process verbally at the start of the interview as well.

Participation from adults with additional cognitive and communication difficulties are welcomed but will require capacity to consent for participation. In care home settings, where potential participants may experience dementia, researchers will check participants' capacity with care staff and only consent those who are able to provide consent independently. For this, the researcher will check that participants can understand the nature of the research, what it entails, be able to retain the information to decide about participation, be able to make a free choice, and be capable of making this decision at a time it needs to be made.

Dissemination of findings

We will prepare and publish articles in appropriate peer-reviewed journals and professional magazines (e.g., British Academy of Audiology (BAA) Audacity) to ensure the findings are shared with audiology healthcare professionals and academics researching hearing loss. Findings will be disseminated via poster and oral presentations at relevant academic and non-scientific conferences and meetings, and social media. They will also be disseminated to PPI groups, audiology services and local commissioners.

Acknowledgements

The study is sponsored by University Hospitals Bristol and Weston NHS Foundation Trust and supported by Aston University Applied Audiology research group.

Funding

This study is supported by a National Institute for Health Research (NIHR) Health Services and Delivery Research programme (Funding stream REF NIHR 131597). The sponsor for this study is the University Hospitals Bristol and Weston NHS Foundation Trust. Aston University (Applied Audiology research group, College of Health and Life Sciences) is the supporting institution. Dr Jonathan Banks

For peer review only - http://bmjopen.bmj.com/site/about/guidelines.xhtml

is partly funded by National Institute for Health and Care Research Applied Research Collaboration West (NIHR ARC West) and NIHR Health and Social Care Delivery HS&DR (REF NIHR 131597).

Competing interests: Authors have no competing interests to declare.

Authors' contributions: HP is the lead researcher leading the protocol development, ethical approval, data collection, data analysis and dissemination. SS wrote the first draft of the protocol and final manuscript. GBOC, RS, SH, JB, AH, RK, RG and JS have provided feedback and support in the development of the protocol and study documents including the ethics application and interview schedule. All authors read, contributed to, edited, and agreed the final manuscript.

Patient consent for publication: N/A

Provenance and peer review: This protocol was reviewed by the South-West Research Design Service Advisory Group; members of the Research Design Service; Aston University Research Governance team; PPI groups and patient experts.

References

1. WHO. Deafness and hearing loss. https://www.who.int/news-room/fact-sheets/detail/deafness-and-hearing-loss (accessed 13 Sep 2022). 2022.

2. RNID. Hearing loss. https://rnid.org.uk/information-and-support/hearing-loss/ (accessed 13 Sep 2022). 2022.

3. Action on Hearing Loss. Hearing Matters: Why urgent action is needed on deafness, tinnitus and hearing loss across the UK. https://rnid.org.uk/about-us/research-and-policy/social-research-reports/hearing-matters/ (date accessed 17 Sep 2022). 2020.

4. Malcolm KA, Suen JJ, Nieman CL. Socioeconomic position and hearing loss: current understanding and recent advances. Current opinion in otolaryngology & head and neck surgery. 2022;30(5):351-7.

For peer review only - http://bmjopen.bmj.com/site/about/guidelines.xhtml

5. Scholes S, Biddulph J, Davis A, Mindell JS. Socioeconomic differences in hearing among middle-aged and older adults: cross-sectional analyses using the Health Survey for England. BMJ open. 2018;8(2):e019615.
6. Barker AB, Leighton P, Ferguson MA. Coping together with hearing loss: a qualitative meta-synthesis of the psychosocial experiences of people with hearing loss and their communication partners. International journal of audiology. 2017;56(5):297-305.
7. Chen HL. Hearing in the elderly: Relation of hearing loss, loneliness, and self-esteem. Journal of Gerontological Nursing. 1994;20(6):22-8.
8. Shukla A, Harper M, Pedersen E, Goman A, Suen JJ, Price C, et al. Hearing Loss, Loneliness, and Social Isolation: A Systematic Review. Otolaryngology--head and neck surgery : official journal of American Academy of Otolaryngology-Head and Neck Surgery. 2020;162(5):622-33.
9. Lawrence BJ, Jayakody DMP, Bennett RJ, Eikelboom RH, Gasson N, Friedland PL. Hearing Loss and Depression in Older Adults: A Systematic Review and Meta-analysis. The Gerontologist. 2020;60(3):e137-e54.
10. Besser J, Stropahl M, Urry E, Launer S. Comorbidities of hearing loss and the implications of multimorbidity for audiological care. Hearing Research. 2018;369:3-14.
11. Lin FR, Metter EJ, O'Brien RJ, Resnick SM, Zonderman AB, Ferrucci L. Hearing Loss and Incident Dementia. Archives of Neurology. 2011;68(2):214-20.
12. Loughrey DG, Kelly ME, Kelley GA, Brennan S, Lawlor BA. Association of Age-Related Hearing Loss With Cognitive Function, Cognitive Impairment, and Dementia: A Systematic Review and Meta-analysis. JAMA Otolaryngology–Head & Neck Surgery. 2018;144(2):115-26.
13. Griffiths TD, Lad M, Kumar S, Holmes E, McMurray B, Maguire EA, et al. How Can Hearing Loss Cause Dementia? Neuron. 2020;108(3):401-12.
14. Ferguson MA, Kitterick PT, Chong LY, Edmondson-Jones M, Barker F, Hoare DJ. Hearing aids for mild to moderate hearing loss in adults. Cochrane Database of Systematic Reviews. 2017(9).
15. Kitterick PT, Ferguson MA. Hearing Aids and Health-Related Quality of Life in Adults With Hearing Loss. JAMA. 2018;319(21):2225-6.
16. Chisolm TH, Johnson CE, Danhauer JL, Portz LJ, Abrams HB, Lesner S, et al. A systematic review of health-related quality of life and hearing aids: final report of the American Academy of Audiology Task Force On the Health-Related Quality of Life Benefits of Amplification in Adults. Journal of the American Academy of Audiology. 2007;18(2):151-83.
17. Dillon H, Day J, Bant S, Munro KJ. Adoption, use and non-use of hearing aids: a robust estimate based on Welsh national survey statistics. International journal of audiology. 2020;59(8):567-73.
18. Davis A, Smith P, Ferguson M, Stephens D, Gianopoulos I. Acceptability, benefit and costs of early screening for hearing disability: a study of potential screening tests and models. Health technology assessment (Winchester, England). 2007;11(42):1-294.
19. Holman JA, Ali YHK, Naylor G. A qualitative investigation of the hearing and hearing-aid related emotional states experienced by adults with hearing loss. International journal of audiology. 2022:1-10.

For peer review only - http://bmjopen.bmj.com/site/about/guidelines.xhtml

20. Wright D, Gagné JP. Acclimatization to Hearing Aids by Older Adults. Ear Hear. 2021;42(1):193-205.

21. Claesen E, Pryce H. An exploration of the perspectives of help-seekers prescribed hearing aids. Primary health care research & development. 2012;13(3):279-84.

22. Humes LE, Wilson DL, Barlow NN, Garner C. Changes in hearing-aid benefit following 1 or 2 years of hearing-aid use by older adults. Journal of speech, language, and hearing research : JSLHR. 2002;45(4):772-82.

23. McCormack A, Fortnum H. Why do people fitted with hearing aids not wear them? International journal of audiology. 2013;52(5):360-8.

24. Stieglitz Ham H, Bunn P, Meyer C, Khan A, Hickson L. Exploring the relationship between technology use, hearing help-seeking, and hearing aid outcomes in older adults. International journal of audiology. 2014;53 Suppl 1:S38-42.

25. May CR, Eton DT, Boehmer K, Gallacher K, Hunt K, MacDonald S, et al. Rethinking the patient: using Burden of Treatment Theory to understand the changing dynamics of illness. BMC Health Services Research. 2014;14(1):281.

26. Gallacher K, May CR, Montori VM, Mair FS. Understanding patients' experiences of treatment burden in chronic heart failure using normalization process theory. Annals of family medicine. 2011;9(3):235-43.

27. Jowsey T, Yen L, W PM. Time spent on health related activities associated with chronic illness: a scoping literature review. BMC Public Health. 2012;12(1):1044.

28. Ferguson M, Brandreth M, Brassington W, Leighton P, Wharrad H. A Randomized Controlled Trial to Evaluate the Benefits of a Multimedia Educational Program for First-Time Hearing Aid Users. Ear Hear. 2016;37(2):123-36.

29. Sweetow RW, Sabes JH. The need for and development of an adaptive Listening and Communication Enhancement (LACE) Program. Journal of the American Academy of Audiology. 2006;17(8):538-58.

30. Spyridakou C, Mendis S, DeVal D. Improving transition outcomes in adolescents with permanent hearing loss. BMJ Open Quality. 2019;8(2):e000336.

31. Department of Health. Operational Guidance to the NHS: extending patient choice of provider. Gateway ref 16242. 2011.

32. Pryce H, Hall A. The role of shared decision-making in audiologic rehabilitation. Perspectives on Aural Rehabilitation and Its Instrumentation. 2014;21(1):15-23.

33. Pryce H, Hall A, Laplante-Lévesque A, Clark E. A qualitative investigation of decision making during help-seeking for adult hearing loss. International journal of audiology. 2016;55(11):658-65.

34. NICE. Hearing loss in adults: assessment and management. NICE guideline Published: 21 June 2018 www.nice.org.uk/guidance/ng98 (accessed 12 Sep 2022). 2018.

35. Barker AB, Leighton P, Ferguson MA. Coping together with hearing loss: a qualitative meta-synthesis of the psychosocial experiences of people with hearing loss and their communication partners. International journal of audiology. 2017;56(5):297-305.

36. Strauss A, Corbin J. Grounded theory methodology: An overview. 1994.

For peer review only - http://bmjopen.bmj.com/site/about/guidelines.xhtml

37. Strauss A, JM. C. Grounded theory in practice: Sage; 1997.

38. Corbin J SA. Basics of qualitative research: Techniques and procedures for developing grounded theory. Sage publications; 2014 Nov 25. 2014.

39. Braun V, Clarke V. To saturate or not to saturate? Questioning data saturation as a useful concept for thematic analysis and sample-size rationales. Qualitative Research in Sport, Exercise and Health. 2021;13(2):201-16.

40. Dawes P, Fortnum H, Moore DR, Emsley R, Norman P, Cruickshanks K, et al. Hearing in middle age: a population snapshot of 40- to 69-year olds in the United Kingdom. Ear Hear. 2014;35(3):e44-51.

41. World Health Organization. World report on hearing. Licence: CC BY-NC-SA 3.0 IGO https://www.who.int/publications/i/item/9789240020481 [Date accessed 13 March 2023]. Geneva; 2021.

For peer review only

For peer review only - http://bmjopen.bmj.com/site/about/guidelines.xhtml

Aston University
Birmingham

NHS
University Hospitals
Bristol and Weston
NHS Foundation Trust

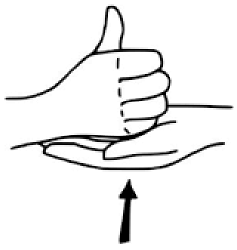

Help

Patient/Participation Participant Information Sheet

Hearing Loss and Patient Reported Experience (HELP) Using patient experience to improve audiology

We need your HELP!

As someone with hearing loss, we would love your help in our study in which we are aiming to learn more about the lived experience of having hearing loss. The help we require would be in the format of an interview and questionnaire, which should take no longer than 1 hour 30 minutes. If this sounds like something you are able to do, please read on.

What is the purpose of the study?

The aim of this study is to understand the patient's experience and the efforts people make to manage their hearing. The information you provide us with will go towards helping us create a questionnaire. This questionnaire is called a Patient Reported Experience Measure. This in turn will help us improve audiology services and improve the practical support we give people with hearing loss.

Why have I been invited??

We would love your participation in our research, as someone seeking help with their hearing and/or experiencing hearing loss.

For peer review only - http://bmjopen.bmj.com/site/about/guidelines.xhtml

Aston University
Birmingham

NHS
University Hospitals Bristol and Weston
NHS Foundation Trust

What will happen if I take part?

If you choose to take part in this study, you may be invited to participate in interviews and then invited to complete some questionnaires, including the Patient Reported Experience Measure.

We need about 30-40 people in total to participate in interviews to tell us about hearing loss. We then need a further 300 people to complete some questionnaires which are designed to capture the experience of hearing loss. Questionnaires can be completed online or on paper. We will include a stamped addressed envelope if you need to post them to us.

We may interview you again after you have completed the questionnaires to ask you what you think of the questionnaires and how we could improve them. However, as we are only recruiting a small number of people to interview after completing the questionnaire you may not be asked to take part in this. We may invite you to participate in all of these activities or you can choose one or the other.

We will conduct individual interviews at a location that suits you. That may be in person at a university or clinic premises or online or over the phone. Unfortunately, we cannot reimburse your travel expenses or time.

We will contact you using the contact details you give us e.g. phone or email.

Do I have to take part?

No, taking part is entirely voluntary. If you decide not to take part or decide later to withdraw from the study, you won't have to give a reason and the care you receive will not be affected. You can withdraw at any time by ringing or writing to us. If you do choose to withdraw, we will only keep the data collected up to the point of withdrawal.

For peer review only - http://bmjopen.bmj.com/site/about/guidelines.xhtml

Aston University
Birmingham

NHS
University Hospitals
Bristol and Weston
NHS Foundation Trust

Are there any risks involved?

No! Participating in the study should not cause you any inconvenience or discomfort. We are asking for your time - up to 1 hour for an interview and, on a different day, another 30 minutes for completing questionnaires.

What are the benefits?

Your experiences are crucial to help us understand what people experience with hearing loss so we can better design the services that give support. Participating in the study will not change your clinical care right now, but might improve it in the future.

How will we keep your data confidential?

The interviews will be recorded, and the audio files will be kept on an encrypted memory stick and a secure computer until they can be transcribed into an anonymous word document. Once it is transcribed the audio file will be deleted. No one other than the researcher and you will know that you have taken part in the study. Your clinical team will not know what you say.

How will we use information about you?

We will need to use information from for this research project.

This information will include your name and contact details. People will use this information to do the research or to check your records to make sure that the research is being done properly. People who do not need to know who you are will not be able to see your name or contact details. Your data will have a code number instead.

We will keep all information about you safe and secure.

Once we have finished the study, we will keep some of the data so we can check the results. We will write our reports in a way that no-one can work out that you took part in the study.

What are your choices about how your information is used?

- You can stop being part of the study at any time, without giving a reason, but we will keep information about you that we already have.

For peer review only - http://bmjopen.bmj.com/site/about/guidelines.xhtml

Aston University
Birmingham

NHS
University Hospitals Bristol and Weston
NHS Foundation Trust

- We need to manage your records in specific ways for the research to be reliable. This means that we won't be able to let you see or change the data we hold about you.

Where can you find out more about how your information is used?

You can find out more about how we use your information

- at www.hra.nhs.uk/information-about-patients/
- our leaflet available from our website www.uhbristol.nhs.uk/research-innovation/for-patients-and-public/how-we-use-your-information-(gdpr)/
- by asking one of the research team
- by sending an email to our Data Protection Officer: InformationGovernance@UHBW.nhs.uk
- by ringing us on 0117 34 23701 or 0117 34 23794. (Data Protection Officer)

Who is organising and funding the project?

This study has been designed by patients, audiology clinicians and researchers. The project is led by a research team at Aston University in Birmingham and the research is funded by the National Institute of Health Research (NIHR) in the Health and Social Care Delivery Research (HS&DR) Programme.

All research in the NHS is looked at by an independent group of people and this study has been reviewed and approved by the relevant Research Ethics and Health Research Authority (HRA) committees.

What do I do now?

If you are interested in taking part in our study, please contact our research team by e-mail or telephone number asgiven below. We will then discuss whether you would like to participate in interviews and questionnaire completion. We will then arrange to meet at a time that is convenient for you.

Who to contact for further information?

For peer review only - http://bmjopen.bmj.com/site/about/guidelines.xhtml

The Chief Investigator is Dr Helen Pryce and you can ask her any questions about the study, including any questions or concerns you may have in taking part.

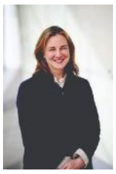

audiology_helpstudy@aston.ac.uk

0121 204 4131

Patient Advice and Liaison Service

If you have concerns about the way you have been approached or treated during the course of the study, you may wish to contact **Patient Advice and Liaison Service**

Kempthorne House,
St Martin's Hospital,
Midford Road,
Bath
BA2 5RPon: **t:** 01225 831500
e: customer services@hcrgcaregroup.com

Thank you for reading this leaflet and considering our research

For peer review only - http://bmjopen.bmj.com/site/about/guidelines.xhtml
For peer review only

COREQ (COnsolidated criteria for REporting Qualitative research) Checklist

A checklist of items that should be included in reports of qualitative research. You must report the page number in your manuscript where you consider each of the items listed in this checklist. If you have not included this information, either revise your manuscript accordingly before submitting or note N/A.

Topic	Item No.	Guide Questions/Description	Reported on Page No.
**Domain 1: Research team and reflexivity**			
Personal characteristics			
Interviewer/facilitator	1	Which author/s conducted the interview or focus group?	
Credentials	2	What were the researcher's credentials? E.g. PhD, MD	
Occupation	3	What was their occupation at the time of the study?	
Gender	4	Was the researcher male or female?	
Experience and training	5	What experience or training did the researcher have?	
Relationship with participants			
Relationship established	6	Was a relationship established prior to study commencement?	
Participant knowledge of the interviewer	7	What did the participants know about the researcher? e.g. personal goals, reasons for doing the research	
Interviewer characteristics	8	What characteristics were reported about the inter viewer/facilitator? e.g. Bias, assumptions, reasons and interests in the research topic	
**Domain 2: Study design**			
Theoretical framework			
Methodological orientation and Theory	9	What methodological orientation was stated to underpin the study? e.g. grounded theory, discourse analysis, ethnography, phenomenology, content analysis	
Participant selection			
Sampling	10	How were participants selected? e.g. purposive, convenience, consecutive, snowball	
Method of approach	11	How were participants approached? e.g. face-to-face, telephone, mail, email	
Sample size	12	How many participants were in the study?	
Non-participation	13	How many people refused to participate or dropped out? Reasons?	
Setting			
Setting of data collection	14	Where was the data collected? e.g. home, clinic, workplace	
Presence of non-participants	15	Was anyone else present besides the participants and researchers?	
Description of sample	16	What are the important characteristics of the sample? e.g. demographic data, date	
Data collection			
Interview guide	17	Were questions, prompts, guides provided by the authors? Was it pilot tested?	
Repeat interviews	18	Were repeat inter views carried out? If yes, how many?	
Audio/visual recording	19	Did the research use audio or visual recording to collect the data?	
Field notes	20	Were field notes made during and/or after the inter view or focus group?	
Duration	21	What was the duration of the inter views or focus group?	
Data saturation	22	Was data saturation discussed?	
Transcripts returned	23	Were transcripts returned to participants for comment and/or	

For peer review only

Topic	Item No.	Guide Questions/Description	Reported on Page No.
		correction?	
**Domain 3: analysis and findings**			
Data analysis			
Number of data coders	24	How many data coders coded the data?	
Description of the coding tree	25	Did authors provide a description of the coding tree?	
Derivation of themes	26	Were themes identified in advance or derived from the data?	
Software	27	What software, if applicable, was used to manage the data?	
Participant checking	28	Did participants provide feedback on the findings?	
Reporting			
Quotations presented	29	Were participant quotations presented to illustrate the themes/findings? Was each quotation identified? e.g. participant number	
Data and findings consistent	30	Was there consistency between the data presented and the findings?	
Clarity of major themes	31	Were major themes clearly presented in the findings?	
Clarity of minor themes	32	Is there a description of diverse cases or discussion of minor themes?	

Developed from: Tong A, Sainsbury P, Craig J. Consolidated criteria for reporting qualitative research (COREQ): a 32-item checklist for interviews and focus groups. *International Journal for Quality in Health Care*. 2007. Volume 19, Number 6: pp. 349 – 357

Once you have completed this checklist, please save a copy and upload it as part of your submission. DO NOT include this checklist as part of the main manuscript document. It must be uploaded as a separate file.

For peer review only

BMJ Open

Protocol for a qualitative study exploring the lived experience of Hearing Loss and Patient Reported Experience in the UK: The HeLP study

Journal:	*BMJ Open*
Manuscript ID	bmjopen-2022-069363.R2
Article Type:	Protocol
Date Submitted by the Author:	05-May-2023
Complete List of Authors:	Pryce, Helen; Aston University College of Health and Life Sciences, School of Optometry<br>Smith, Sian; Aston University College of Health and Life Sciences, School of Optometry<br>Burns-O'Connell, Georgina; Aston University College of Health and Life Sciences, School of Optometry<br>Shaw, Rachel; Aston University School of Psychology; Aston University College of Health and Life Sciences, Aston Institute of Health & Neurodevelopment<br>Hussain, Saira; Aston University College of Health and Life Sciences, Department of Audiology<br>Banks, Jonathan; National Institute for Health Research, Applied Research Collaboration West, University Hospitals Bristol and Weston NHS Foundation Trust; University of Bristol Medical School, Population Health Sciences<br>Hall, Amanda; Aston University College of Health and Life Sciences, School of Optometry; University Hospitals Bristol NHS Foundation Trust, Children's Hearing Centre<br>Knibb, Rebecca; Aston University, School of Psychology<br>Greenwood, Rosemary; University Hospitals Bristol and Weston NHS Foundation Trust<br>Straus, Jean; NIHR ARC Northwest London,
**Primary Subject Heading**:	Qualitative research
Secondary Subject Heading:	Health services research, Ear, nose and throat/otolaryngology
Keywords:	QUALITATIVE RESEARCH, Quality in health care < HEALTH SERVICES ADMINISTRATION & MANAGEMENT, Audiology < OTOLARYNGOLOGY, Patient-Centered Care

SCHOLARONE™
Manuscripts

Protocol for a qualitative study exploring the lived experience of Hearing Loss and Patient Reported Experience in the UK: The HeLP study

Authors: The HeLP project team

Dr Helen Pryce [1] *
Dr Sian K Smith [1]
Dr Georgina Burns O'Connell [1]
Dr Rachel Shaw [2, 3]
Saira Hussain [4]
Dr Jonathan Banks [5 6]
Dr Amanda Hall [1 7]
Dr Rebecca Knibb [2]
Dr Rosemary Greenwood [7]
Jean Straus [8]

Corresponding author: Dr Helen Pryce, College of Life and Health Sciences, Aston University, Birmingham, UK, B4 7ET; Email: h.pryce-cazalet@aston.ac.uk

[1] School of Optometry, College of Life and Health Sciences, Aston University, Birmingham, UK, B4 7ET
[2] School of Psychology, Aston University, Birmingham, UK, B4 7ET
[3] Institute of Health and Neurodevelopment, Aston University, Birmingham, UK, B4 7ET
[4] Department of Audiology, College of Health and Life Sciences, Birmingham, B4 7ET, UK
[5] National Institute for Health Research, Applied Research Collaboration West (NIHR ARC West), University Hospitals Bristol and Weston NHS Foundation Trust, Bristol, UK, BS1 2NT
[6] Population Health Sciences, Bristol Medical School, University of Bristol, Bristol, UK, BS8 2BN
[7] University Hospitals Bristol and Weston NHS Foundation Trust, Bristol, UK, BS1 3NU
[8] Patient and Public Involvement Representative and Lead, HeLP study, UK

For peer review only

For peer review only - http://bmjopen.bmj.com/site/about/guidelines.xhtml

ABSTRACT

Introduction

Worldwide, hearing loss is a significant public health issue and one of the most common chronic health conditions experienced by older adults. Hearing loss is associated with communication difficulties, social withdrawal, isolation, and lower quality of life. Although hearing aid technology has improved significantly, the workload of managing hearing aids has increased. The aim of this qualitative study is to develop a novel theory of people's lived experience of hearing loss across the lifespan.

Methods

Eligible participants will be young people and adults aged 16 years and above who have a hearing loss and carers/ family members of people with a hearing loss. This study will use individual, in-depth face-to-face or online interviews. With participants' permission, interviews will be audio-recorded and transcribed verbatim. A grounded theory approach to concurrent data gathering and analysis will develop grouped codes and categories and link these to provide a novel theory to describe the experience of hearing loss.

Ethics and dissemination

The study was approved by the West of Scotland Research Ethics Service (approval date: 6 May 2022 ref: 22/WS/0057) and the Health Research Authority and Health and Care Research Wales (HCRW) Approval (approval date:14 June 2022; IRAS project ID: 308816). The research will inform the development of a Patient Reported Experience Measure (PREM) to improve the information and support given to patients. Findings will be disseminated through peer-reviewed articles and at academic conferences, as well as to our Patient and Public Involvement (PPI) groups, healthcare professionals, audiology services and local commissioners.

Keywords: qualitative research, grounded theory, hearing loss, patient experience, hearing aids, help-seeking

Word count: 3998

For peer review only

Strengths and limitations of this study

- Our qualitative interview study, informed by grounded theory approaches, will generate a novel theory on the lived experience of hearing loss across the life course.
- Different patient, public involvement (PPI) groups will be involved throughout the project to guide the logistics of conducting the research and support data analysis procedures.
- Purposive sampling via different clinical sites and non-clinical routes (e.g., lip reading classes, social media) will help to ensure diverse experiences and views are captured.
- The study will be restricted to people living in the UK which may limit the applicability of the findings to other settings.
- This qualitative study part of a wider study that will inform the development of the first Patient Reported Experience Measure (PREM) internationally to enable audiologists to identify the individualised and effective support.

For peer review only

INTRODUCTION

Hearing loss is a chronic health condition that affects an estimated 430 million adults worldwide (1). With the ageing population, hearing loss is set to affect 700 million (one in ten) of the global population by 2050. In the UK, hearing loss affects around 12 million (one in five) adults, which is predicted to increase to 14.2 million by 2035 (2). The prevalence of hearing loss rises sharply with age. In the UK, around 40% of adults aged over 50 years old live with hearing loss, increasing to 70% of older adults over the age of 70 (3). Hearing loss perpetuates socioeconomic inequality with its greatest prevalence associated with lower socioeconomic status (4, 5).

The implications of hearing loss are well-established and far reaching. People with hearing loss may feel ashamed or frustrated about their difficulty communicating and limit their social interactions (6). This separation can lead to social isolation, and feelings of loneliness, anxiety, depression, as well as fewer opportunities for engaging in listening and communicating (7-10). Hearing loss is linked with other chronic health conditions, including arthritis, cancer, cardiovascular risk factors, diabetes, stroke, visual impairment, and mobility issues (11). There is also growing evidence that hearing loss increases the risk of developing dementia, although the mechanisms linking the two are as yet unclear (12-14).

There is no permanent cure for hearing loss, and the main form of clinical intervention is wearing hearing aids. Using hearing aids has been shown to improve hearing function for mild to moderate hearing loss, as well as positively influence quality of life through enhancing communication (15-17). Despite these benefits, hearing aid use is low and approximately 30% of people report wearing hearing aids some of the time and 20% do not wear them at all (18). Furthermore, many people live with hearing loss symptoms for a long time (on average, up to 10 years) before seeking help (19). Understanding the reasons why hearing aid usage is low and gaining insight into why people do not seek help are important research priorities.

The illness burden of hearing loss is considerable. Hearing loss makes it hard work to communicate and increases risks in all aspects of life (6, 20). Adjusting to hearing aids can be particularly tiring and frustrating as it takes time to adapt to amplified sounds and to learn how amplified sounds correspond to previous auditory memories (21). All the treatment work is devolved to the patient, including wearing the device each day, and replacing the fine tubing monthly, the batteries weekly, keeping the aid at the right volume settings. The stigma about visible deficits combined with the association of age with wearing hearing aids often means that people do not want hearing aids (22-25). Although hearing aid technology and assistive devices have advanced, the workload of managing hearing aids has increased significantly (e.g., learning how to use Bluetooth to pair devices, sequencing turning on and off different devices) (26). Hearing aid non-use (including people who do not have hearing aids and those who have hearing aids but find them difficult to manage) has been attributed as a problem of the hearing aid user, their motivation or ability, rather than a reflection of an onerous workload. The

For peer review only - http://bmjopen.bmj.com/site/about/guidelines.xhtml

trade-off between the benefits of wearing hearing aids versus the work required to use them is likely to vary across the life course.

The ‘burden of treatment’ theory is an increasingly useful way to consider the relative benefits of allocating health resources (27). Health services systematically transfer work to patients to manage long-term conditions (28, 29). Previous research has focussed on giving patients more to do, e.g. education and skills training (30, 31) which further enhances inequality as not all adults are equally able to undertake the work required (5). Work with younger people with hearing loss transitioning into adult audiology services emphasises the burden in navigating audiology services (32). Similarly, in older age, the benefits of hearing aids may be outweighed by the burden of work required to access and use them (22). This work is out of view of audiology services and thus overlooked when planning how to provide care.

In the UK, audiology services are commissioned differently in different parts of the country, with the introduction of ‘Any Qualified Provider’ (AQP) services meaning that large corporations and optical chains have been awarded NHS contracts to provide audiology services in England (33). AQP service providers are primarily funded to provide hearing aids, so there is little time (if any) to identify patients’ values and to engage in shared decision making about preferred treatment options (34, 35). At the same time, guidelines from the National Institute of Clinical Excellence (NICE) propose that patients be fitted with hearing aids and then given motivational interviewing to increase their motivation to use them (36). More experiential evidence is needed to understand the reasons why people do and do not use hearing aids and the factors that encourage their use.

It is critical that service development and delivery are grounded in experiential evidence to develop a good understanding of the daily illness and treatment burdens of hearing. Previous work shows that while there are benefits in aspects of communication from hearing aids, there are specific barriers to use that derive from social identity preservation and stigma of hearing loss (and hearing aids as a visible marker of hearing loss) (20, 22, 35, 37). Our own work suggests that people seek help with hearing to gain information and support rather than hearing aids per se (Claesen & Pryce, 2012). However, changes in commissioning of audiology services have resulted in patients being prescribed hearing aids rather than engaging in shared decision making (Pryce et al, 2016).

Further research is required to understand how people make the trade-offs at different life stages between living with a hearing loss and using a hearing aid to manage the hearing loss. Such research will enable audiology services to be sensitive to patient experience and promote shared decision making. Therefore, the overall goal of the current study is to provide a deeper understanding of the lived experiences of coping with hearing loss (with and without hearing aids) at different stages of life, from the perspectives of patients and their families. This study forms part of a larger study – the Hearing Loss and Patient Reported Experience study (HeLP study) – the first study of its kind to prioritise patients’ lived experience of hearing loss to inform decision-making in audiology care

For peer review only - http://bmjopen.bmj.com/site/about/guidelines.xhtml

planning and services. The findings from this qualitative work will inform the development of potential items for a novel Patient Reported Experience Measure (PREM) to be used in clinical practice to understand the work involved in living with hearing loss ('illness work') and hearing aids ('treatment work').

Aims and objectives

Drawing on grounded theory methodology, the aim of this study is to develop a comprehensive novel theory that has the potential to explain variation in people's lived experiences of hearing loss. The objectives are to:

1. Develop a theoretical understanding of how coping with hearing loss ('illness work') and accessing and living with hearing aids ('treatment work') is experienced and negotiated alongside personal and socio-cultural contexts.
2. Understand how individuals adapt to and manage hearing aids.
3. Explore the reasons why hearing aid non-use occurs.
4. Explore patients' experiences of accessing audiology services and support.

METHODS AND ANALYSIS

Study design

This qualitative interview study will be informed by Strauss and Corbin's pragmatic approach to grounded theory (38, 39). This allows for (and recognises the likelihood of) researchers to bringing prior knowledge to the process of refining theoretical concepts. Grounded theory methodologies are particularly well suited to developing an understanding of patterns across contrasting cases and processes that influence those patterns. In comparison to other qualitative methodologies, grounded theories offer explanatory potential as well as description (40). In this work, we want to identify the work involved in hearing loss and hearing aid use, how it is experienced, and the factors informing variation in this experience. This study will be conducted and reported in accordance with the consolidated criteria for reporting qualitative research checklist (COREQ) (41), and the Guideline for Reporting and Evaluating Grounded Theory Research Studies (GUREGT) (42).

Research team and reflexivity

For reflexivity, the study team comprises two female clinician-researchers (HP – an academic and hearing therapist with clinical experience in audiological rehabilitation and grounded theory methodology; SH – a clinical scientist, teaching fellow and researcher in audiology), three female researchers (GBOH – a teaching fellow and researcher with a background in sociology and interest in hearing research; RS – a Health Psychologist with experience of applied qualitative research; SS – a Health Psychologist with personal interest in hearing research) and a female PPI lead (JS) who is an expert in audiology by experience and writer. All the team either have first-hand experience of hearing loss or are closely related to someone who does.

For peer review only - http://bmjopen.bmj.com/site/about/guidelines.xhtml

Participants will be interviewed by either HP, GBOC, SH or SS. At the start of each interview, the interviewers will undertake a 'working alliance' discussion agreeing participant and researcher roles, timing and nature of the interview. This will include the researcher's interest and motivations in the topic.

The study team will meet fortnightly to reflect on their role in shaping data collection and compare and contrast their interpretations of the data. Throughout the study, the wider HeLP research group and core PPI members will discuss interpretations of the findings for triangulation and work together to develop and link theoretical categories for the explanatory model.

Study steering committee group

The Study Steering Committee will have overall oversight of the study and provide advice to the study research team, funder, and sponsor. Our steering group will comprise 8 independent members, including: 1 international collaborative representative, 2 experts by experience, 3 clinicians working in audiology in different parts of the country, 1 social science researcher and chaired by a health commissioner with a particular interest in person centred care. The steering group will meet every 6 months and oversee delivery milestones and reporting.

Study setting

The study is being conducted in the UK with participants (adults with hearing loss and carers, family members or parents) recruited from three clinical sites in England (Bristol and Bath) and Scotland (Tayside) and non-clinical groups (e.g., lip reading classes, social care settings).

Participants and recruitment

Participants are young people and adults aged 16 years and over and individuals who have experience of hearing loss directly or as a carer, partner or parent. Eligibility criteria (Box 1) are intentionally broad to recruit participants of different ages across the life course as it is acknowledged that hearing loss experience and needs will vary at different life stages. The geographical spread of the different clinical sites (rural, urban, and semi-urban) will enable us to achieve socioeconomic variation and find cases across the age range with contrasting features in terms of sex, income, housing, and clinical needs.

Drawing on the principles of Strass and Corbin's grounded theory approach, we will be using purposive sampling to provide maximum variation of cases to generate rich data from different perspectives which will allow us to target under-represented groups. We will examine the experience of different age groups and identify similarities and differences between and across the life stages This will enable us to build up an understanding of living with hearing loss (with and without hearing aids) across the life course.

For peer review only - http://bmjopen.bmj.com/site/about/guidelines.xhtml

Potentially eligible participants will be made aware of the study through advertisements. Clinical sites will advertise the study to new and existing patients via word-of-mouth, invitation flyers or posters displayed in waiting/reception areas. Participants will also be recruited through non-clinical routes including care homes, supported living centres and community groups. Advertisements will also be distributed via leads of tinnitus groups, teachers of lip-reading classes, and staff in residential care settings. Details of the study will be circulated on social media (e.g., Twitter) and the study link hosted on Aston university website with the aim of recruiting participants with diverse experiences and backgrounds. In addition, our Patient and Public Involvement (PPI) collaborator in South Asian community groups and care homes will prompt snowball sampling in which word of mouth advertisement will encourage potential participants to contact the study team. In accordance with our PPI group in the South Asian community regarding recruitment of participants who are not help seekers, we will recruit participants from local mosques and women's groups in the local area (e.g., exercise groups for older Asian women and mindfulness group for Asian women. Previous research experience informed our sample estimate of approximately 25-38 patients and 8-12 family members/carers (see Box 1) to capture a range of experiences and achieve sufficient information power, but this will be reviewed as data collection progresses.

Interested participants will directly contact the HeLP study team via email, phone, or post. The researchers will then provide potential participants with the participant information sheet (online supplemental file). Informed consent will be obtained from researchers trained in Good Clinical Practice (GCP) and experienced with research. Recruitment commenced June 2022 continuing through April 2023. At the time of writing, 18 participants have been recruited and data collection is ongoing.

Box 1. Eligibility criteria for study participation and sample

Inclusion criteria

- Young adults (aged 16-29 years): Transitioning from paediatric to adult services & negotiating independence. We will aim to recruit 4-6 young people, including those with additional disabilities such as learning disabilities, and up to 4 parents.

- Adults aged 30-49 years: Managing hearing loss while pursuing career/work and family life; 4-6 people with diagnosed hearing loss either using or not using hearing aids, plus 2-4 partners/relatives.

- Adults aged 50-79 years: Noticing symptoms of hearing loss for the first time; 4-6 hearing aid users and 5-8 non hearing aid users.

- Adults aged 80 years to end of life: Most likely to have hearing loss. Other health conditions likely and changes in living situation possible; 4-6 hearing aid users, 4-6 non hearing aid users and 2-4 carers from residential care settings.

For peer review only - http://bmjopen.bmj.com/site/about/guidelines.xhtml

Exclusion criteria

- Volunteers without close connection to or experience of hearing loss.

Data collection

Data will be collected using qualitative, semi-structured interviews by the study team (GBOC,HP, SS,SH) who are all experienced in qualitative research. Participants will have the option for the interview to take place at their preferred location including online (via Microsoft Teams), or at their home, the university or audiology department in a quiet and private space. An interview schedule been developed to explore the lived experience of managing hearing loss and hearing service use (Box 2). It has been informed by a literature review, the expertise of the authors and input from the PPI groups. It covers: motivations to take part in the study, lived experience of hearing loss, challenges of hearing loss (emotions, practical efforts), the journeys and processes of referral and diagnosis, experiences of audiology services and support, decision-making around whether to wear hearing aids and trade-offs made between managing with and without hearing aids. Open-ended questions will be used to encourage the participant to talk in-depth and flexibly about their experiences (Box 2). The expected duration of the interviews is around 1 hour. With participants' permission, the interviews will be recorded on a digital voice recorder or online (video and audio-recorded) using Microsoft Teams and supplemented with field notes to capture important information not captured by the audio-recording. We will loan specialist headsets to enable people with hearing loss to hear better on the phone and via online platforms. All transcripts will be anonymised, and pseudonyms will be used. Transcripts will not be returned to participants for comment and/or correction as we want to capture experiences and views at that particular moment in time.

Box 2. Interview questions for patients and carers/ family members

Questions for people with hearing loss

- Tell me a little bit about yourself
- Tell me your story with your hearing and why you were interested in taking part?
- Can you tell me your thoughts and feelings about hearing loss?
- What have you found difficult about having a hearing loss?
- What have you done to manage your hearing loss?
- Decision making around hearing aid use or non-use/ uptake – Tell me how you came to be using/not using your assistive listening devices/hearing aids explore reasons why/why not?
- What is important to you when deciding whether to use hearing devices/aids?
- Who else is important in helping you decide?
- Has this changed over time?
- What has been helpful to you?
- Have you sought help from audiology services?
- Tell me about your experience with using audiology services
- Based on your experience – what do you think they ought to know/ do that they currently don't do?

Questions for family/ carers

- Tell me what is it like for you managing hearing loss?
- What is difficult for you about living with someone with hearing loss?
- What if anything do you do differently as a result of hearing loss?

For peer review only - http://bmjopen.bmj.com/site/about/guidelines.xhtml

- Have you had any involvement with audiology services?
- What was that like?
- What is challenging about using hearing aids (if your family member/cared for person has them)?

Data analysis

In line with Strauss and Corbin's grounded theory procedures, data analysis and interviewing will be iterative, in which early analysis and findings will be compared to inform ongoing interviews. We will keep field notes of reflections and thoughts following interviews to support this. Whilst the notion of data 'saturation' has been widely critiqued as a neo-positivist indication of a singular 'truth'; there is recognition of the importance of capturing a range of views, based on different contexts (43). The research team will review the data until we are confident that a wide range of experiences are captured and there is sufficient conceptual depth to generate meaningful insights to address the research aims (43). Meaning statements will be examined and every line of transcript will be coded by three coders (GBOC, SH, SS) to summarise key content (open coding). Codes will be compared across and between transcripts and will be collated into broader categories with properties and dimensions that capture the range of meanings (axial coding). Finally, the relationships between these categories will be explored to develop a conceptual model that explains the variation in experience (selective coding). Triangulation is a core part of our activity with multiple researcher and PPI members contributing a view to the interpretation which is justified by each researcher. We will consider core categories that offer explanatory potential to the paradigm and explain variation within the data set (40). Our analysis will describe the range of 'illness work' of hearing loss and the 'treatment work' of using hearing aids and audiology services. We will also capture the variations that exist and the important influences that contrast different social, cultural and age groups.

Rigour in analysis

We will ensure credibility to the research process through transparent decision making, including incorporating PPI views. The lead investigator (HP) and co-investigator (RS) will supervise the analysis closely and ensure consistency. They will blind code a subset of transcripts and compare coding. The HeLP qualitative research team (GBOC, HP, SH, JS, SS) will develop and refine the conceptual model whilst interviews and analysis progress to understand the effort required to manage hearing loss and highlight the variations in patient need at each life stage. We will examine the fit of this model to previous models and existing theories, and we will encourage reflection from all researchers and PPI leads through discussion.

Patient and public involvement (PPI)

A major component of the HeLP study is the involvement of PPI groups. Specifically, two PPI leads (a researcher with PPI responsibility and a public member) will manage and coordinate the PPI activities, working closing with the study team. Our PPI leads have experience in communicating with

For peer review only - http://bmjopen.bmj.com/site/about/guidelines.xhtml

people with complex communication needs. PPI activities include reviewing patient information sheets and consent forms, advising on recruitment and interview questions, checking analysis procedures, and addressing any uncertainties in data interpretation We will consult our PPI groups to check that our process and early findings have not omitted any aspects of the experience (e.g. labelling codes and categories, development of conceptual framework). Fieldnote diaries will record PPI engagement and outcomes.

Certain groups are more likely to be affected by hearing loss including people from South Asia (Indian, Pakistani, and Bangladeshi communities), older adults in residential care and adults with learning disabilities. Our PPI strategy reflects the diverse and heterogeneous nature of the population affected by hearing loss in the UK. We have directly targeted the following groups to expand our reach of PPI inclusion:

1. South Asian community groups – we have interest and agreement from these groups. We have also spoken to local religious centres, including but not limited to specific local Imams to enhance access to those from Muslim backgrounds. South Asian heritage people have both a disproportionately high risk of hearing loss and are underrepresented in patient populations in the UK (44, 45).

2. Residential care homes – our PPI leads will meet with individuals in these settings (with whom we have existing relationships) face-to-face.

3. Aston PPI group – people in the local community who have experience with hearing loss, a group including student members who are younger (18-40). They meet virtually and will advise via whatever form of communication suits them best (e.g., phone, email, online messaging).

4. Bath PPI group – a well-established group of older adults who support and advise on service delivery and research. They meet face-to-face or via email.

Our initial PPI work informed the development of the proposal in a number of ways. Firstly, our Aston group highlighted the need for researchers from the same culture and religious as participants to undertake PPI and research. As such, we will include a researcher with responsibility for PPI in marginalised groups and have recruited a researcher dedicated to South Asian groups. Secondly, our older group identified problems with hearing aids that inform the notion of ‘work’ (e.g. struggles to fit hearing aids, learning skills such as battery changing). These issues will be explored further by targeting PPI and recruitment in residential care settings where access to audiology services can be challenging. Thirdly, our older group noted the need to be flexible in where and how data gathering occurs and be mindful of travel and distance barriers for participants. This informed our plans to gather data in people’s homes, at clinics, university, and online/ telephone. Finally, our younger

For peer review only - http://bmjopen.bmj.com/site/about/guidelines.xhtml

group advised we purchase amplifier headsets which could be sent out to participants for online/phone use, if required.

ETHICS AND DISSEMINATION

Ethical approval

This study was reviewed and approved by the West of Scotland Research Ethics Service (approval date: 6 May 2022 ref: 22/WS/0057) and the Health Research Authority and Health and Care Research Wales (HCRW) Approval (approval date:14 June 2022; IRAS project ID: 308816).

Participant withdrawal

Each participant has the right to withdraw at any time during the study and information relating to all withdrawals will be recorded. If a participant wishes to discontinue, data collected up until that point will be kept and included in the analysis.

Confidentiality

All data obtained from participants will be kept confidential. Participants will be anonymised with all identifying information removed. Participants' names will be replaced with pseudonyms.

Benefits to participation

Participants may reflect on their experience of hearing loss (as a person living with hearing loss or as a carer/ family member) and gain new understandings which might be empowering. Their insights will help inform how to better design audiology services and improve clinical practice and care.

Assessment and management of risks

There is a possibility that discussing some topics will create distress. If at any point a participant feels distressed during the interview, they will be given the option to pause the recording and provide time to discuss the issue. The researcher will signpost participants to other services including patient liaison services within NHS providers. In the case of home interviews, researchers will follow the sponsor's (University Hospitals Bristol and Weston NHS Trust) lone working policy including plans to check in with another member of the research team before and after interviews.

Storage of data

All study data will be stored on the university password protected server. Informed consent sheets (hard copies) containing personal information will be kept in a secure filing cabinet at the university only accessible to study staff and authorised personnel. Audio recordings of interviews will be conducted with encrypted digital recorders or MS Teams recording software. Once transcribed verbatim and checked for accuracy, recordings will be deleted. Data will be collected and retained in accordance with the Data Protection Act 2018 and General Data Protection Regulation (GDPR) standards. Once data analysis is completed, the study documents (paper and electronic) will be

For peer review only - http://bmjopen.bmj.com/site/about/guidelines.xhtml

retained in a secure location for 5 years at Aston University. Personal data (e.g. contact details) are held on a separate Aston University Box file and are separate from research data. Besides clinicians in Participant Identification Centres (PICs) sending the advertisements to home addresses we will not access personal data.

Informed consent

We will obtain written informed consent from all interview participants at the start of an arranged interview time. Participants will have had at least a week to consider the participant information sheet and ask questions in advance. Participants who volunteer for online meetings will post their consent form back in a pre-paid envelope. The researcher will record the consent process verbally at the start of the interview as well.

Participation from adults with additional cognitive and communication difficulties are welcomed but will require capacity to consent for participation. In care home settings, where potential participants may experience dementia, researchers will check participants' capacity with care staff and only consent those who are able to provide consent independently. For this, the researcher will check that participants can understand the nature of the research and be able to retain the information to make a free choice about participation, and be capable of making this decision at a time it needs to be made.

Dissemination of findings

We will prepare and publish articles in appropriate peer-reviewed journals and professional magazines (e.g., British Academy of Audiology (BAA) Audacity) to ensure the findings are shared with audiology healthcare professionals and academics. Findings will be disseminated via poster and oral presentations at relevant academic and non-scientific conferences, and social media. They will also be disseminated to PPI groups, audiology services and local commissioners.

Acknowledgements

The sponsor for this study is the University Hospitals Bristol and Weston NHS Foundation Trust. Aston University (Applied Audiology research group, College of Health and Life Sciences) is the supporting institution.

Funding

This study is supported by a National Institute for Health Research (NIHR) Health Services and Delivery Research programme (Funding stream REF NIHR 131597). Dr Jonathan Banks is partly funded by National Institute for Health and Care Research Applied Research Collaboration West (NIHR ARC West) and NIHR Health and Social Care Delivery HS&DR (REF NIHR 131597).

For peer review only - http://bmjopen.bmj.com/site/about/guidelines.xhtml

Competing interests: Authors have no competing interests to declare.

Authors' contributions: HP is the lead researcher leading the protocol development, ethical approval, data collection, data analysis and dissemination. SS wrote the first draft of the protocol, the final manuscript and prepared the submission. GBOC, RS, SH, JB, AH, RK, RG and JS have provided feedback and support in the development of the protocol and study documents including the ethics application and interview schedule. All authors read, contributed to, edited, and agreed the final manuscript.

Patient consent for publication: N/A

Provenance and peer review: This protocol was reviewed by the South-West Research Design Service Advisory Group; members of the Research Design Service; Aston University Research Governance team; PPI groups and patient experts.

For peer review only

References

1. WHO. Deafness and hearing loss. https://www.who.int/news-room/fact-sheets/detail/deafness-and-hearing-loss (accessed 13 Sep 2022). 2022.

2. RNID. Hearing loss. https://rnid.org.uk/information-and-support/hearing-loss/ (accessed 13 Sep 2022). 2022.

3. Action on Hearing Loss. Hearing Matters: Why urgent action is needed on deafness, tinnitus and hearing loss across the UK. https://rnid.org.uk/about-us/research-and-policy/social-research-reports/hearing-matters/ (date accessed 17 Sep 2022). 2020.

4. Malcolm KA, Suen JJ, Nieman CL. Socioeconomic position and hearing loss: current understanding and recent advances. Current opinion in otolaryngology & head and neck surgery. 2022;30(5):351-7.

5. Scholes S, Biddulph J, Davis A, Mindell JS. Socioeconomic differences in hearing among middle-aged and older adults: cross-sectional analyses using the Health Survey for England. BMJ open. 2018;8(2):e019615.

For peer review only - http://bmjopen.bmj.com/site/about/guidelines.xhtml

6. Barker AB, Leighton P, Ferguson MA. Coping together with hearing loss: a qualitative meta-synthesis of the psychosocial experiences of people with hearing loss and their communication partners. International journal of audiology. 2017;56(5):297-305.
7. Chen HL. Hearing in the elderly: Relation of hearing loss, loneliness, and self-esteem. Journal of Gerontological Nursing. 1994;20(6):22-8.
8. Shukla A, Harper M, Pedersen E, Goman A, Suen JJ, Price C, et al. Hearing Loss, Loneliness, and Social Isolation: A Systematic Review. Otolaryngology--head and neck surgery : official journal of American Academy of Otolaryngology-Head and Neck Surgery. 2020;162(5):622-33.
9. Lawrence BJ, Jayakody DMP, Bennett RJ, Eikelboom RH, Gasson N, Friedland PL. Hearing Loss and Depression in Older Adults: A Systematic Review and Meta-analysis. The Gerontologist. 2020;60(3):e137-e54.
10. Laird EC, Bennett RJ, Barr CM, Bryant CA. Experiences of Hearing Loss and Audiological Rehabilitation for Older Adults With Comorbid Psychological Symptoms: A Qualitative Study. Am J Audiol. 2020;29(4):809-24.
11. Besser J, Stropahl M, Urry E, Launer S. Comorbidities of hearing loss and the implications of multimorbidity for audiological care. Hearing Research. 2018;369:3-14.
12. Lin FR, Metter EJ, O'Brien RJ, Resnick SM, Zonderman AB, Ferrucci L. Hearing Loss and Incident Dementia. Archives of Neurology. 2011;68(2):214-20.
13. Loughrey DG, Kelly ME, Kelley GA, Brennan S, Lawlor BA. Association of Age-Related Hearing Loss With Cognitive Function, Cognitive Impairment, and Dementia: A Systematic Review and Meta-analysis. JAMA Otolaryngology–Head & Neck Surgery. 2018;144(2):115-26.
14. Griffiths TD, Lad M, Kumar S, Holmes E, McMurray B, Maguire EA, et al. How Can Hearing Loss Cause Dementia? Neuron. 2020;108(3):401-12.
15. Ferguson MA, Kitterick PT, Chong LY, Edmondson-Jones M, Barker F, Hoare DJ. Hearing aids for mild to moderate hearing loss in adults. Cochrane Database of Systematic Reviews. 2017(9).
16. Kitterick PT, Ferguson MA. Hearing Aids and Health-Related Quality of Life in Adults With Hearing Loss. JAMA. 2018;319(21):2225-6.
17. Chisolm TH, Johnson CE, Danhauer JL, Portz LJ, Abrams HB, Lesner S, et al. A systematic review of health-related quality of life and hearing aids: final report of the American Academy of Audiology Task Force On the Health-Related Quality of Life Benefits of Amplification in Adults. Journal of the American Academy of Audiology. 2007;18(2):151-83.
18. Dillon H, Day J, Bant S, Munro KJ. Adoption, use and non-use of hearing aids: a robust estimate based on Welsh national survey statistics. International journal of audiology. 2020;59(8):567-73.
19. Davis A, Smith P, Ferguson M, Stephens D, Gianopoulos I. Acceptability, benefit and costs of early screening for hearing disability: a study of potential screening tests and models. Health technology assessment (Winchester, England). 2007;11(42):1-294.
20. Holman JA, Ali YHK, Naylor G. A qualitative investigation of the hearing and hearing-aid related emotional states experienced by adults with hearing loss. International journal of audiology. 2022:1-10.

For peer review only - http://bmjopen.bmj.com/site/about/guidelines.xhtml

21. Wright D, Gagné JP. Acclimatization to Hearing Aids by Older Adults. Ear Hear. 2021;42(1):193-205.

22. Claesen E, Pryce H. An exploration of the perspectives of help-seekers prescribed hearing aids. Primary health care research & development. 2012;13(3):279-84.

23. Humes LE, Wilson DL, Barlow NN, Garner C. Changes in hearing-aid benefit following 1 or 2 years of hearing-aid use by older adults. Journal of speech, language, and hearing research : JSLHR. 2002;45(4):772-82.

24. McCormack A, Fortnum H. Why do people fitted with hearing aids not wear them? International journal of audiology. 2013;52(5):360-8.

25. Knudsen LV, Nielsen C, Kramer SE, Jones L, Laplante-Lévesque A. Client Labor: Adults with Hearing Impairment Describing Their Participation in Their Hearing Help-Seeking and Rehabilitation. Journal of the American Academy of Audiology. 2013;24(03):192-204.

26. Stieglitz Ham H, Bunn P, Meyer C, Khan A, Hickson L. Exploring the relationship between technology use, hearing help-seeking, and hearing aid outcomes in older adults. International journal of audiology. 2014;53 Suppl 1:S38-42.

27. May CR, Eton DT, Boehmer K, Gallacher K, Hunt K, MacDonald S, et al. Rethinking the patient: using Burden of Treatment Theory to understand the changing dynamics of illness. BMC Health Services Research. 2014;14(1):281.

28. Gallacher K, May CR, Montori VM, Mair FS. Understanding patients' experiences of treatment burden in chronic heart failure using normalization process theory. Annals of family medicine. 2011;9(3):235-43.

29. Jowsey T, Yen L, W PM. Time spent on health related activities associated with chronic illness: a scoping literature review. BMC Public Health. 2012;12(1):1044.

30. Ferguson M, Brandreth M, Brassington W, Leighton P, Wharrad H. A Randomized Controlled Trial to Evaluate the Benefits of a Multimedia Educational Program for First-Time Hearing Aid Users. Ear Hear. 2016;37(2):123-36.

31. Sweetow RW, Sabes JH. The need for and development of an adaptive Listening and Communication Enhancement (LACE) Program. Journal of the American Academy of Audiology. 2006;17(8):538-58.

32. Spyridakou C, Mendis S, DeVal D. Improving transition outcomes in adolescents with permanent hearing loss. BMJ Open Quality. 2019;8(2):e000336.

33. Department of Health. Operational Guidance to the NHS: extending patient choice of provider. Gateway ref 16242. 2011.

34. Pryce H, Hall A. The role of shared decision-making in audiologic rehabilitation. Perspectives on Aural Rehabilitation and Its Instrumentation. 2014;21(1):15-23.

35. Pryce H, Hall A, Laplante-Lévesque A, Clark E. A qualitative investigation of decision making during help-seeking for adult hearing loss. International journal of audiology. 2016;55(11):658-65.

36. NICE. Hearing loss in adults: assessment and management. NICE guideline Published: 21 June 2018 www.nice.org.uk/guidance/ng98 (accessed 12 Sep 2022). 2018.

For peer review only - http://bmjopen.bmj.com/site/about/guidelines.xhtml

37. Barker AB, Leighton P, Ferguson MA. Coping together with hearing loss: a qualitative meta-synthesis of the psychosocial experiences of people with hearing loss and their communication partners. International journal of audiology. 2017;56(5):297-305.

38. Strauss A, Corbin J. Grounded theory methodology: An overview. 1994.

39. Strauss A, JM. C. Grounded theory in practice: Sage; 1997.

40. Corbin J SA. Basics of qualitative research: Techniques and procedures for developing grounded theory. Sage publications; 2014 Nov 25. 2014.

41. Tong A, Sainsbury P, Craig J. Consolidated criteria for reporting qualitative research (COREQ): a 32-item checklist for interviews and focus groups. International Journal for Quality in Health Care. 2007;19(6):349-57.

42. Berthelsen CB, Grimshaw-Aagaard SLS, Hansen C. Developing a Guideline for Reporting and Evaluating Grounded Theory Research Studies (GUREGT). International Journal of Health Sciences,. 2018;6(1):64-76.

43. Braun V, Clarke V. To saturate or not to saturate? Questioning data saturation as a useful concept for thematic analysis and sample-size rationales. Qualitative Research in Sport, Exercise and Health. 2021;13(2):201-16.

44. Dawes P, Fortnum H, Moore DR, Emsley R, Norman P, Cruickshanks K, et al. Hearing in middle age: a population snapshot of 40- to 69-year olds in the United Kingdom. Ear Hear. 2014;35(3):e44-51.

45. World Health Organization. World report on hearing. Licence: CC BY-NC-SA 3.0 IGO https://www.who.int/publications/i/item/9789240020481 [Date accessed 13 March 2023]. Geneva; 2021.

For peer review only

For peer review only - http://bmjopen.bmj.com/site/about/guidelines.xhtml

Aston University
Birmingham

NHS
University Hospitals
Bristol and Weston
NHS Foundation Trust

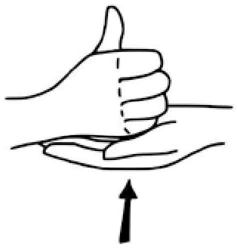

Help

Patient/Participation Participant Information Sheet

Hearing Loss and Patient Reported Experience (HELP) Using patient experience to improve audiology

For peer review only

We need your HELP!

As someone with hearing loss, we would love your help in our study in which we are aiming to learn more about the lived experience of having hearing loss. The help we require would be in the format of an interview and questionnaire, which should take no longer than 1 hour 30 minutes. If this sounds like something you are able to do, please read on.

What is the purpose of the study?

The aim of this study is to understand the patient's experience and the efforts people make to manage their hearing. The information you provide us with will go towards helping us create a questionnaire. This questionnaire is called a Patient Reported Experience Measure. This in turn will help us improve audiology services and improve the practical support we give people with hearing loss.

Why have I been invited??

We would love your participation in our research, as someone seeking help with their hearing and/or experiencing hearing loss.

For peer review only - http://bmjopen.bmj.com/site/about/guidelines.xhtml

Aston University
Birmingham

NHS
University Hospitals
Bristol and Weston
NHS Foundation Trust

What will happen if I take part?

If you choose to take part in this study, you may be invited to participate in interviews and then invited to complete some questionnaires, including the Patient Reported Experience Measure.

We need about 30-40 people in total to participate in interviews to tell us about hearing loss. We then need a further 300 people to complete some questionnaires which are designed to capture the experience of hearing loss. Questionnaires can be completed online or on paper. We will include a stamped addressed envelope if you need to post them to us.

We may interview you again after you have completed the questionnaires to ask you what you think of the questionnaires and how we could improve them. However, as we are only recruiting a small number of people to interview after completing the questionnaire you may not be asked to take part in this. We may invite you to participate in all of these activities or you can choose one or the other.

We will conduct individual interviews at a location that suits you. That may be in person at a university or clinic premises or online or over the phone. Unfortunately, we cannot reimburse your travel expenses or time.

We will contact you using the contact details you give us e.g. phone or email.

Do I have to take part?

No, taking part is entirely voluntary. If you decide not to take part or decide later to withdraw from the study, you won't have to give a reason and the care you receive will not be affected. You can withdraw at any time by ringing or writing to us. If you do choose to withdraw, we will only keep the data collected up to the point of withdrawal.

For peer review only - http://bmjopen.bmj.com/site/about/guidelines.xhtml

Aston University
Birmingham

NHS
University Hospitals
Bristol and Weston
NHS Foundation Trust

Are there any risks involved?

No! Participating in the study should not cause you any inconvenience or discomfort. We are asking for your time - up to 1 hour for an interview and, on a different day, another 30 minutes for completing questionnaires.

What are the benefits?

Your experiences are crucial to help us understand what people experience with hearing loss so we can better design the services that give support. Participating in the study will not change your clinical care right now, but might improve it in the future.

How will we keep your data confidential?

The interviews will be recorded, and the audio files will be kept on an encrypted memory stick and a secure computer until they can be transcribed into an anonymous word document. Once it is transcribed the audio file will be deleted. No one other than the researcher and you will know that you have taken part in the study. Your clinical team will not know what you say.

How will we use information about you?

We will need to use information from for this research project.

This information will include your name and contact details. People will use this information to do the research or to check your records to make sure that the research is being done properly. People who do not need to know who you are will not be able to see your name or contact details. Your data will have a code number instead.

We will keep all information about you safe and secure.

Once we have finished the study, we will keep some of the data so we can check the results. We will write our reports in a way that no-one can work out that you took part in the study.

What are your choices about how your information is used?

- You can stop being part of the study at any time, without giving a reason, but we will keep information about you that we already have.

For peer review only - http://bmjopen.bmj.com/site/about/guidelines.xhtml

- We need to manage your records in specific ways for the research to be reliable. This means that we won't be able to let you see or change the data we hold about you.

Where can you find out more about how your information is used?

You can find out more about how we use your information

- at www.hra.nhs.uk/information-about-patients/
- our leaflet available from our website www.uhbristol.nhs.uk/research-innovation/for-patients-and-public/how-we-use-your-information-(gdpr)/
- by asking one of the research team
- by sending an email to our Data Protection Officer: InformationGovernance@UHBW.nhs.uk
- by ringing us on 0117 34 23701 or 0117 34 23794. (Data Protection Officer)

Who is organising and funding the project?

This study has been designed by patients, audiology clinicians and researchers. The project is led by a research team at Aston University in Birmingham and the research is funded by the National Institute of Health Research (NIHR) in the Health and Social Care Delivery Research (HS&DR) Programme.

All research in the NHS is looked at by an independent group of people and this study has been reviewed and approved by the relevant Research Ethics and Health Research Authority (HRA) committees.

What do I do now?

If you are interested in taking part in our study, please contact our research team by e-mail or telephone number asgiven below. We will then discuss whether you would like to participate in interviews and questionnaire completion. We will then arrange to meet at a time that is convenient for you.

Who to contact for further information?

For peer review only - http://bmjopen.bmj.com/site/about/guidelines.xhtml

Aston University
Birmingham

NHS
University Hospitals
Bristol and Weston
NHS Foundation Trust

The Chief Investigator is Dr Helen Pryce and you can ask her any questions about the study, including any questions or concerns you may have in taking part.

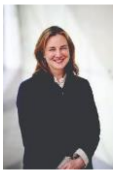

audiology_helpstudy@aston.ac.uk

0121 204 4131

Patient Advice and Liaison Service

If you have concerns about the way you have been approached or treated during the course of the study, you may wish to contact **Patient Advice and Liaison Service**

Kempthorne House,
St Martin's Hospital,
Midford Road,
Bath
BA2 5RPon: **t:** 01225 831500
e: customer services@hcrgcaregroup.com

Thank you for reading this leaflet and considering our research

For peer review only

For peer review only - http://bmjopen.bmj.com/site/about/guidelines.xhtml
